# Activatable theranostic prodrug scaffold with tunable drug release rate for sequential photodynamic and chemotherapy

**DOI:** 10.1002/smo.20230024

**Published:** 2024-02-20

**Authors:** Si‐Yu Wang, Ying‐Hao Pan, Yu‐Chen Qu, Xiao‐Xiao Chen, Na Shao, Li‐Ya Niu, Qing‐Zheng Yang

**Affiliations:** ^1^ Key Laboratory of Radiopharmaceuticals College of Chemistry Beijing Normal University Beijing China; ^2^ Department of Organic Chemistry University of Geneva Geneva Switzerland

**Keywords:** combinational therapy, fluorescent probes, photodynamic therapy, prodrugs, theranostic agents

## Abstract

Glutathione (GSH)‐activated prodrugs are promising for overcoming the limitations of conventional anti‐tumor drugs. However, current GSH‐responsive disulfide groups exhibit unregulated reactivity, making it impossible to precisely control the drug release rate. We herein report a series of GSH‐responsive prodrugs with a “three‐in‐one” molecular design by integrating a fluorescence report unit, stimuli‐responsive unit and chemodrug into one scaffold with tunable aromatic nucleophilic substitution (S_N_Ar) reactivity. The drug release rate of these prodrugs is tailored by modification of substituent groups with different electron‐withdrawing or ‐donating abilities on the BODIPY core. Furthermore, the prodrugs self‐assemble in water to form nanoparticles that serve as photosensitizers to produce reactive oxygen species upon irradiation for photodynamic therapy (PDT). The PDT process also increases the concentration of GSH in cells, further promoting the release of drugs for chemotherapy. This strategy provides a powerful platform for sequential photodynamic and chemotherapy with tunable drug release rates and synergistic therapeutic effects.

## Graphical abstract

We report a series of GSH‐activated prodrugs with tunable drug release rates by the control of aromatic nucleophilic substitution (S_N_Ar) reactivity. The prodrugs self‐assembled in water to form nanoparticles that serve as photosensitizers, allowing a sequential photodynamic‐chemo therapy in cancer cells.

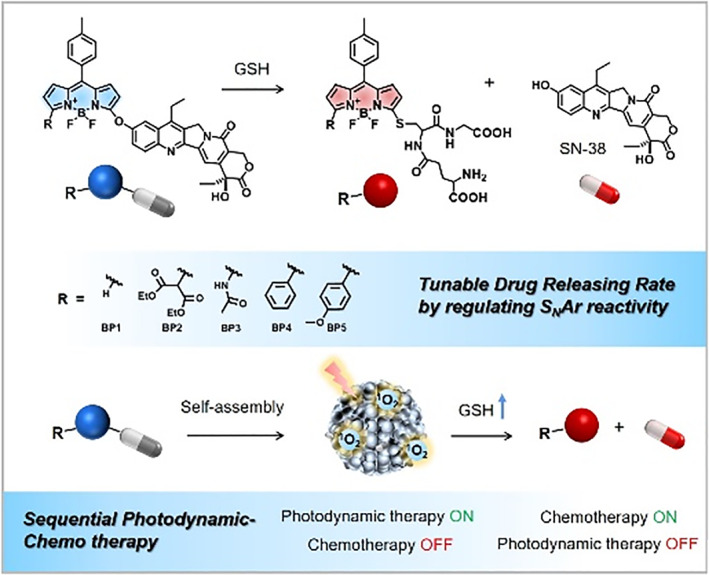

## 1 INTRODUCTION

**SCHEME 1:**
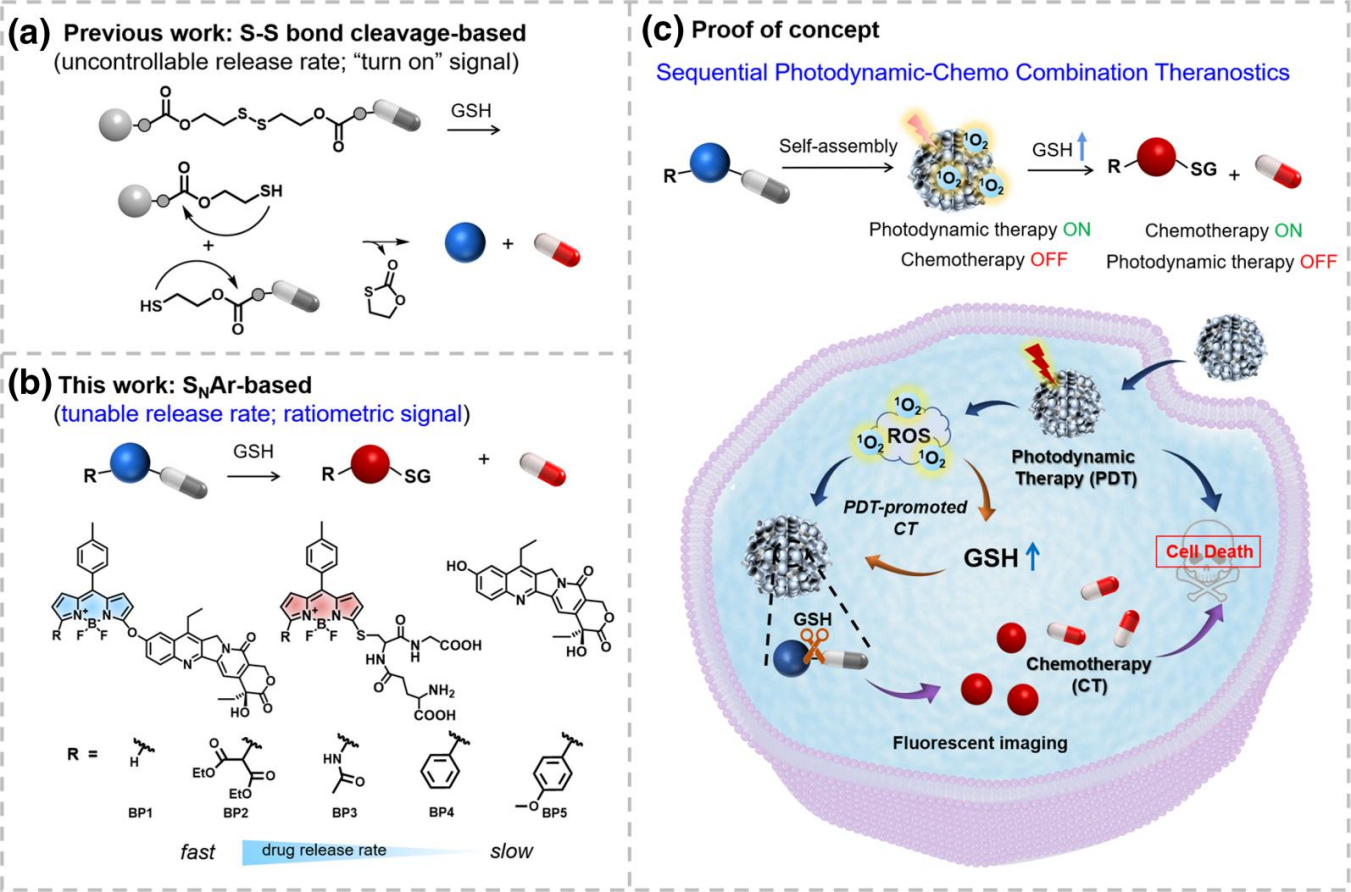
(a) Reaction mechanism of the conventional disulfide‐based prodrugs: the drug release rate is uncontrollable through thiol‐triggered disulfide bond cleavage. (b) GSH‐responsive prodrugs with tunable aromatic nucleophilic substitution (S_N_Ar) reactivity. (c) Schematic illustration of sequential photodynamic‐chemo combination of theranostic prodrugs.

Chemotherapy is one of the most applied clinical therapies in cancer treatment.^[1]^ However, conventional anti‐tumor drugs still face several limitations, such as poor bioavailability, rapid clearance during circulation, and side effects on normal tissues.[^2^, ^3^] Prodrugs are a series of chemically modified pharmacologically active agents that should be transformed in situ to release active drugs.[^4^, ^5^, ^6^, ^7^, ^8^] Theranostic prodrugs are particularly attractive due to their dual functions that offer both therapeutic promise and potential for concurrent diagnosis.[^9^, ^10^, ^11^] Typically, the drug molecule is linked to an imaging reporter with a cleavable linker, allowing the conversion of the non‐cytotoxic prodrug to the cytotoxic drug, as well as the production of readily monitored imaging signals upon being activated. In general, such systems can be activated by tumor‐specific microenvironments (such as pH, reactive oxygen species (ROS), overexpressed enzymes, *etc.*), resulting in a selective anticancer effect.[^12^, ^13^, ^14^, ^15^, ^16^, ^17^, ^18^, ^19^, ^20^, ^21^] Glutathione (GSH) has a concentration of 2–10 mM in cancer cells, which is higher than in normal cells, making it favorable as an activator for selective chemotherapy. Most of the reported GSH‐activated prodrugs use disulfide bonds as reactive linkers (Scheme 1a).[^22^, ^23^, ^24^] However, it is impossible to precisely control the drug release rate because disulfide bond exhibited identical reactivity toward GSH. Therefore, the development of a GSH‐responsive drug delivery system with a tunable drug release rate is attractive for precise drug delivery.^[25]^ However, it has not been achieved yet due to the lack of a reactive linker.

On the other hand, the combination of multiple therapy modalities holds a promising prospect for more efficient cancer treatment. Being an emerging cancer treatment method, photodynamic therapy (PDT) has recently received much attention due to its minimal invasiveness, great spatiotemporal accuracy and low side effects.[^26^, ^27^, ^28^, ^29^, ^30^] It has been proved that the combination of chemotherapy and PDT is an encouraging strategy to cooperatively boost anti‐tumor efficiency and simultaneously minimize side effects of cancer treatment.[^31^, ^32^, ^33^] The conventional way for combination treatment is the simultaneous co‐delivery of anti‐cancer drugs and photosensitizers in one carrier.[^34^, ^35^, ^36^, ^37^] In this case, some problems might be encountered, such as the possibility of drug–drug interactions and between‐drug differences in pharmacokinetic profiles and molecular targets. Instead, sequential treatments provide advantages over simultaneous delivery, potentially reducing adverse effects while maximizing the therapeutic efficacy.[^38^, ^39^] However, how to integrate multiple functions into one prodrug for sequential combination treatments remains a tremendous challenge.

Herein, we design and synthesize a series of GSH‐responsive prodrugs **BP1‐5**, containing GSH‐activated BODIPY as a drug carrier and anti‐tumor drug 7‐ethyl‐10‐hydroxycamptothecin (SN38) as a leaving group. The drug release rate is regulated by the modification of substituent groups on the BODIPY core (Scheme 1). As proof of the concept, GSH‐responsive nanoprodrugs were constructed for sequential photodynamic and chemotherapy. Initially, **BP** molecules self‐assemble in water to form nano‐prodrugs that serve as photosensitizers to produce ROS upon irradiation for PDT. Sequentially, GSH with a high concentration in cancer cells attacks BODIPY by nucleophilic substitution reactions, resulting in the release of SN38, and the GSH‐substituted BODIPY exhibits a ratiometric response, enabling real‐time monitoring of the drug release process. In addition, since oxidative stress defense and tolerance are inherent in tumor cells, cancer cells will further upregulate antioxidant substances[^40^, ^41^, ^42^, ^43^] and improve the antioxidant capacity under the effect of phototherapy. This process of redox remodeling increases the instant GSH concentrations in cells, further promoting the release of drugs. The reaction of **BP** molecules with a high concentration of GSH generates water‐soluble **BP‐SG** that are prone to disaggregate into free molecules, which deactivates ROS generation. Therefore, this process can realize the intelligent control of ROS generation in tumor after PDT treatment. This strategy provides a powerful platform for sequential photodynamic and chemotherapy with tunable drug release rates and synergistic therapeutic effect.

## 2 RESULTS AND DISCUSSIONS

### 2.1 Design principle of “three‐in‐one” theranostic prodrugs with tunable drug release rate

In our previous study, we reported an aromatic nucleophilic substitution‐rearrangement (S_N_Ar‐rearrangement) mechanism based on monochlorinated boron dipyrromethene (BODIPY) for selective detection of biothiols.[^44^, ^45^, ^46^] This mechanism is attractive because not only it provides a general strategy for designing fluorescent probes to detect GSH over Cys/Hcy, but also the reaction kinetics can be controlled by regulation of electron‐donating or ‐withdrawing substituent groups on the BODIPY cores.[^47^, ^48^] We anticipated it could serve as an effective drug carrier for the construction of GSH‐responsive prodrugs. Therefore, five activatable theranostic prodrugs with a “three‐in‐one” molecular design by integrating a luminescent unit, stimuli‐responsive unit and chemodrug into one probe were customized. BODIPY derivatives **BP1‐5** were synthesized and characterized by ^1^H and ^13^C nuclear magnetic resonance and high‐resolution mass spectrometry.

### 2.2 Spectral response of prodrugs toward GSH in solutions

**FIGURE 1:**
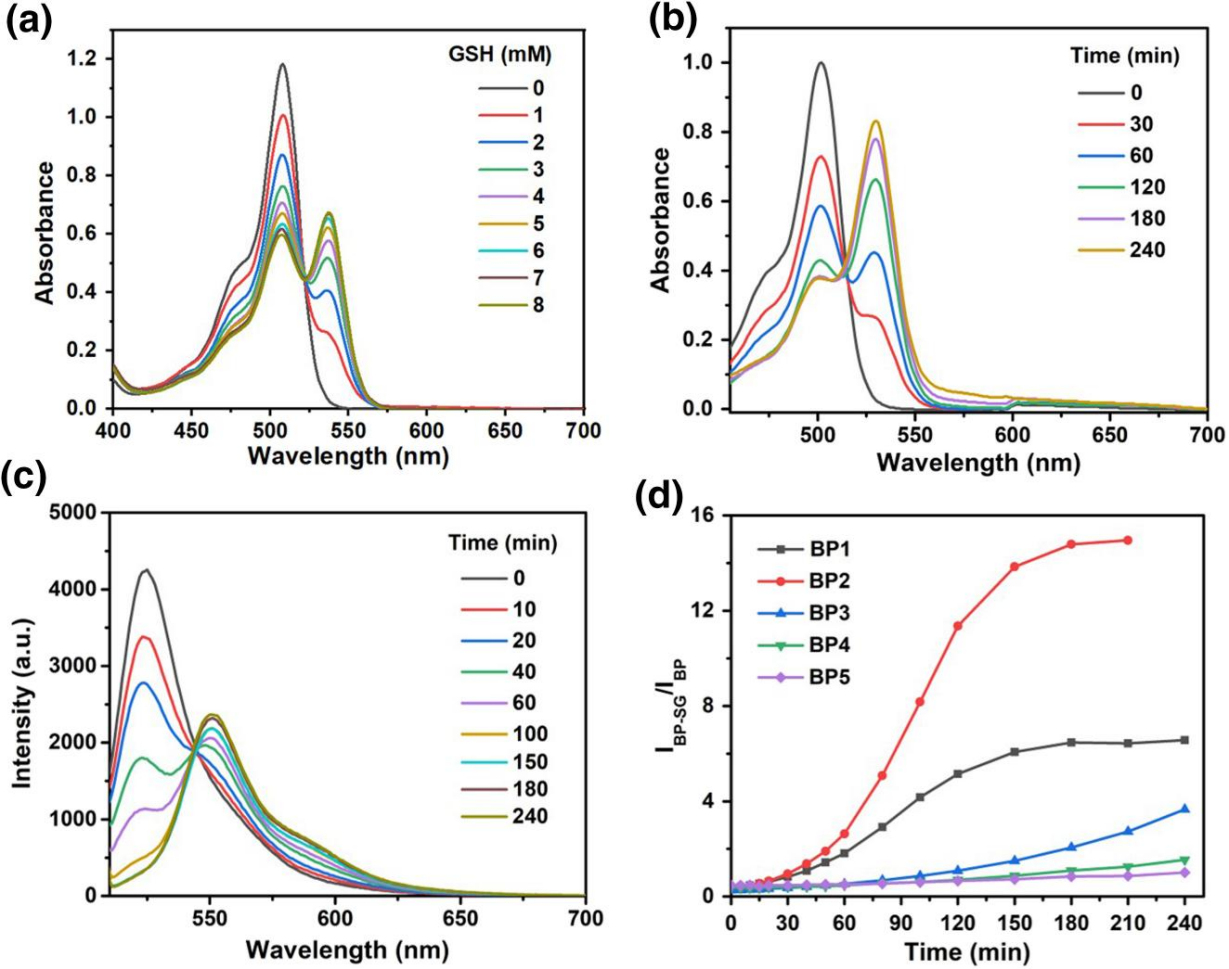
(a) Absorption spectra of 10 μM **BP1** upon addition of 0–8 mM GSH in PBS (10 mM, containing 30 vol% CH_3_CN) at 37°C; (b) Time‐dependent absorption spectra of 10 μM **BP1** treated with 1 mM GSH; (c) Time‐dependent fluorescence spectra of 10 μM **BP1** treated with 1 mM GSH; (d) Ratio of maximum fluorescence intensities of **BP1‐5** before and after the reaction with GSH as a function of time. GSH, Glutathione; PBS, phosphate buffered saline.

Initially, to confirm that the prodrugs could be activated by GSH, the spectral response of the prodrugs in the presence of GSH was investigated. As shown in Figure 1, upon the addition of GSH, a decrease in the original absorption peak of **BP1** at 501 nm was accompanied by the appearance of a new peak at 531 nm (Figure 1a). Similarly, after the reaction with GSH, the maximum absorption peak of each **BP** molecule was red‐shifted by 20–30 nm with apparent isosbestic points, suggesting the formation of **BP‐SG** (Figure S1). From the time‐dependent absorption spectra of **BP** molecules (Figure 1b and Figure S2), the spectra of **BP1** and **BP2** changed most significantly within 2 h, indicating that the reaction rates of **BP1** and **BP2** with GSH are significantly faster than those of **BP3‐5.** The response of **BP** molecules toward GSH was then examined by fluorescence spectroscopy. The fluorescence maximum of each **BP** molecule was red‐shifted by 20–30 nm upon the addition of GSH (Figure 1c and Figure S3), which was in accordance with the absorption spectra. To investigate the selectivity and specific reaction of prodrugs with GSH, the effects of other biothiols, ions and amino acids were also studied. Figure S4 shows that only GSH induced a significant spectra change of prodrugs. Some other thiols such as Cys and Hcy also induced certain fluorescence increases. In contrast, other reagents showed minimal effects, indicating that prodrugs had good selectivity for GSH. The release of SN38 was also monitored by high‐pressure liquid chromatography (HPLC) analysis (Figure S5).

By comparing the time‐dependent fluorescence intensity ratio (I_BP‐SG_/I_BP_), the reactions of **BP1‐2** with GSH were almost completed after ∼ 2 h, while the fluorescence ratio of **BP3‐5** was still increasing even after 4 h (Figure 1d). In HPLC analysis (Figure S5), the drug release efficiency of **BP1** and **BP2** reached up to ∼100% after 60 and 40 min addition of GSH, respectively. By contrast, after the addition of GSH for 96 h, the drug release efficiencies of **BP3‐5** were 42%, 31% and 16%, respectively. We also investigated the ratio of the maximum absorption intensity of **BP** molecules before and after the reaction (A_BP‐SG_/A_BP_). As shown in Figure S6, dose‐dependent signal increases in approximately 60‐fold and 72‐fold were observed when **BP1‐2** was incubated with 1 mM GSH for 10 h, while **BP3‐5** were only partially activated. According to the mechanism of nucleophilic substitution reaction of GSH to BODIPY, these changes could be attributed to the fact that electron‐rich substituents on the 3‐position of BODIPY core decrease the reactivity of nucleophilic aromatic substitution. Therefore, the rate of SN38 release can be regulated by modulating the electron‐withdrawing/donating ability of the BODIPY 3‐position substituent.

### 2.3 Design principle of sequential photodynamic and chemotherapy

**FIGURE 2:**
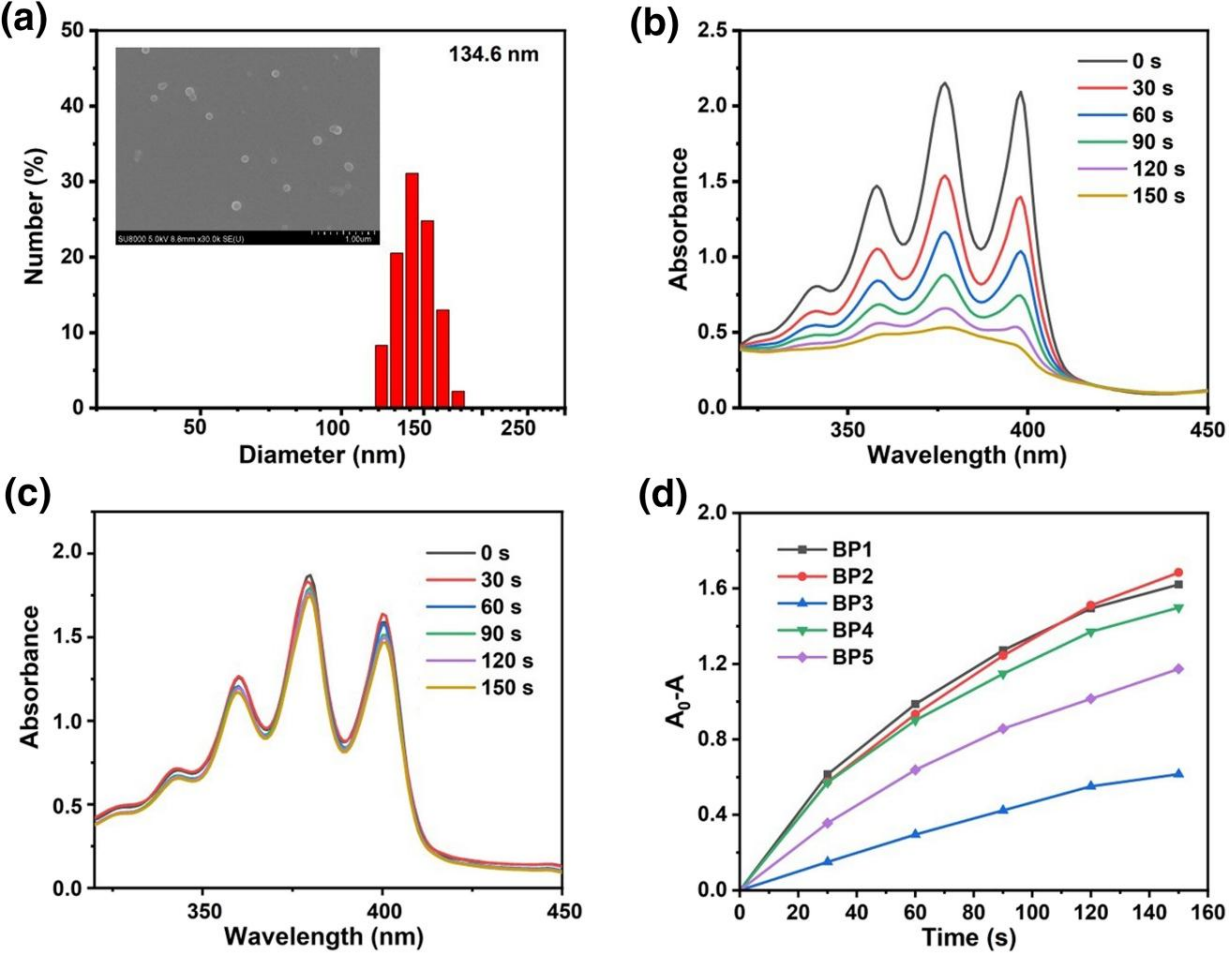
(a) Size distribution of **BP1 NPs** using DLS (inset: SEM image of **BP1 NPs**); (b) Absorption spectra of ABDA in the presence of aqueous dispersion of **BP1 NPs** upon light irradiation for different times; (c) Absorption spectra of ABDA and the reaction mixture of aqueous dispersion of **BP1 NPs** with GSH (5 mM) for 24 h upon light irradiation. (d) Plots of A/A_0_ of ABDA at 378 nm in the presence of **BP1‐5** upon irradiation for different time intervals. ABDA, 9,10‐anthracenediyl‐bis(methylene)‐dimalonic acid; DLS, dynamic light scattering; GSH, Glutathione.

In our previous work, we proposed a supramolecular approach to turn BODIPY fluorophores into photosensitizers for PDT.^[49]^ For a BODIPY monomer without heavy atom, it is difficult for the singlet state to transition to the triplet state because there is a large energy difference between them. Therefore, the monomer cannot react with oxygen to produce singlet oxygen. In contrast, in aggregate states, the energy gaps between relevant excited singlet and triplet states are reduced, leading to considerably improved intersystem‐crossing efficiency. Thus, the nanoparticles exhibit enhanced singlet oxygen generation. In order to obtain effective photosensitizers, the nanoparticles of **BP** molecules, named as **BP NPs**, were prepared by direct self‐assembly of compound **BP** in water under ultrasonication. The SEM images showed that **BP1‐5 NPs** had a regular spherical morphology and the average size of **BP1‐5 NPs** was 120–130 nm estimated by dynamic light scattering (Figure 2a and Figure S7). Singlet oxygen (^1^O_2_) generation was measured with 9,10‐anthracenediyl‐bis(methylene)‐dimalonic acid (ABDA) as the singlet oxygen scavenger. As illustrated in Figure 2b and Figure S8, in the presence of **BP1‐5 NPs**, the characteristic absorption peak of ABDA had a significantly decreased intensity upon white LED light irradiation (Figure 2d). We calculated the ^1^O_2_ yields (*Φ*_Δ_) of **BP1‐5 NPs** using Rose Bengal as a reference, and the resulting values ranged from 0.30 to 0.58 (Figure S8). By contrast, **BP1‐5** in an organic solution caused almost no attenuation of the ABDA absorption. The results suggested that **BP1‐5** had no ^1^O_2_‐generating ability as monomers, while aggregation of **BP1‐5** greatly improved the ^1^O_2_ generation efficiency and realized the successful conversion of fluorophores into photosensitizers. In addition, after the reaction with GSH, the change in ABDA absorption was negligible (Figure 2c). This is because the resultant **BP1‐SG** is water‐soluble, which barely generates ^1^O_2_. It is worth noting that because of the limitation of the traditional “always‐on” photosensitizers, patients are required to remain in the dark for a long period of time (6–8 weeks) after PDT treatment to avoid the undesired photodamage to normal tissues.^[50]^ An effective strategy to rapidly deactivate PDT action after PDT treatment is of great interest both for scientific research and clinical trials. In this way, the side effects of photosensitizer after PDT treatment can be avoided, thus improving the safety of PDT.

### 2.4 Ratiometric imaging of drug release in living cells

**FIGURE 3:**
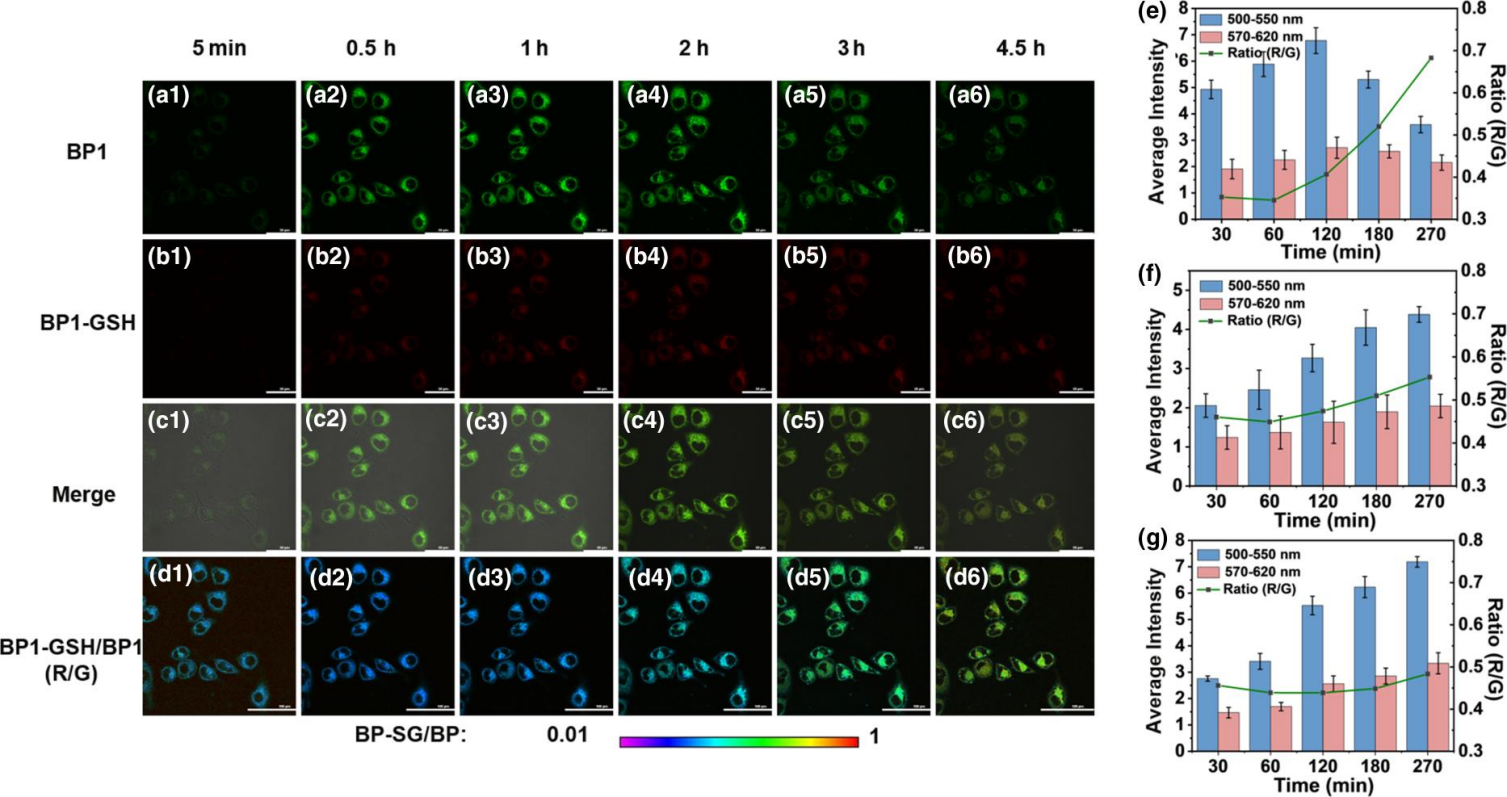
(a–d) Confocal fluorescence image of HeLa cell incubated with **BP1** for different times; (a) **BP1** channel; (b) **BP1‐SG** channel; (c) Merge; (d) Ratio of **BP1‐SG** channel to **BP1** channel; (e–g) Average fluorescence intensity of (e) **BP‐1**, (f) **BP‐2** and (g) **BP‐3** in green (**BP** channel Em: 500–550 nm), red (**BP‐SG** channel Em: 570–620 nm) and ratio channels at different time points. Ex: 487 nm. Scale bar: 100 μm.

To verify the feasibility of **BP** molecules with different substituents regulating the rate of SN38 release in living cells, we monitored the response of **BP** molecules in HeLa cells by confocal fluorescence imaging. HeLa cells were incubated with 10 μM **BP1‐3** followed by real‐time monitoring of the changes in the fluorescence. Clear fluorescence in both green (500–550 nm) and red (570–620 nm) channels was observed, which were assigned to the emission of **BP** and **BP‐SG**, respectively. As shown in Figure 3a,b, with the prolonged incubation time, the fluorescence intensity of the green channel and red channel increased at the beginning and then decreased, with the highest signal intensities at 2 h. These observations suggested that **BP1** was continuously taken up by the cells, and then further reacted with endogenous GSH to form **BP1‐SG**. The weakened signals from **BP1** and **BP1‐SG** suggested that they might be cleared by cellular metabolic pathways. Meanwhile, the average fluorescence intensity ratio between the red channel and the green channel (R/G) increased (Figure 3e), and the growth also accelerated after 1 h, which was ascribed to the increasing conversion of **BP1** to **BP1‐SG** during the reaction. Additionally, we compared the response of different **BP** molecules with GSH (Figure S9 and S10), a gradual increase in the R/G value of **BP1** and **BP2** after 1 h was observed, while that of **BP3** only increased slightly after 3 h, indicating that the reaction rate of **BP3** with endogenous GSH in cells was significantly slower than that of **BP1** and **BP2** (Figures 3f,g). The results in living cells were in accordance with those in solutions. Therefore, the SN38‐release rate was modulated in living cells, and the release process was monitored by real‐time fluorescence imaging of the reaction process between prodrugs and GSH.

### 2.5 Photodynamic therapy in cancer cells

**FIGURE 4:**
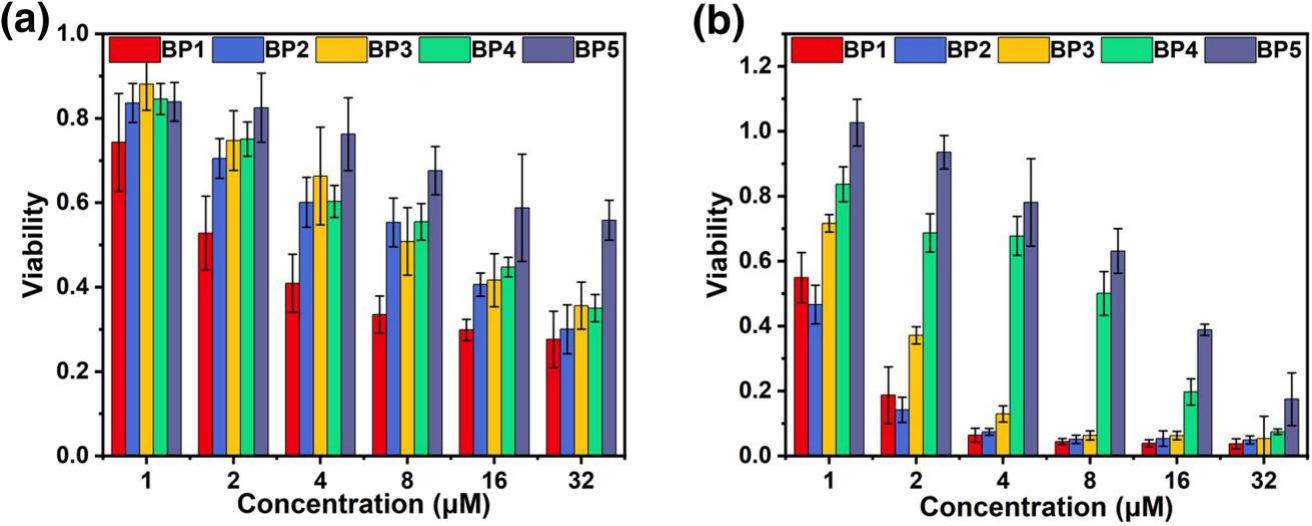
Cell viability of HeLa cells subjected to a range of **BP1‐5** (a) in the dark and (b) upon light irradiation (White LED light, 40 mW/cm^2^).

To verify the chemotherapeutic and photodynamic efficacy of **BP1‐5 NPs** in cancer cells, we validated their cytotoxicity by the cell counting kit‐8 (CCK‐8) assay (Figure 4 and Figure S11). First, we evaluated the dark toxicity. The results showed that they inhibited cell proliferation in a dose‐dependent manner when incubated for 24 h in the absence of light irradiation. Among them, **BP1** showed the best dark toxicity with a half‐maximal inhibitory concentration (IC_50_) of 2.2 μM. Under irradiation with white LED light (40 mW/cm^2^) for 10 min and the same incubation for 24 h, a significant reduction in cell viability could be observed compared to non‐light conditions. In particular, **BP1‐3** showed maximum efficacy enhancement. At an average concentration of 4 μM, cell viability remains above 50% without irradiation and decreases to 10% under irradiation. All these results demonstrated that, in dark conditions, SN38 released after the uptake of **BP** molecules by cells has chemotherapeutic effects; meanwhile, after irradiation, the PDT ability of **BP NPs** and the sequentially activated chemotherapeutic effects synergistically enhanced the cancer cell inhibition efficiency.

### 2.6 Photodynamic therapy promotes release efficiency of prodrug

**FIGURE 5:**
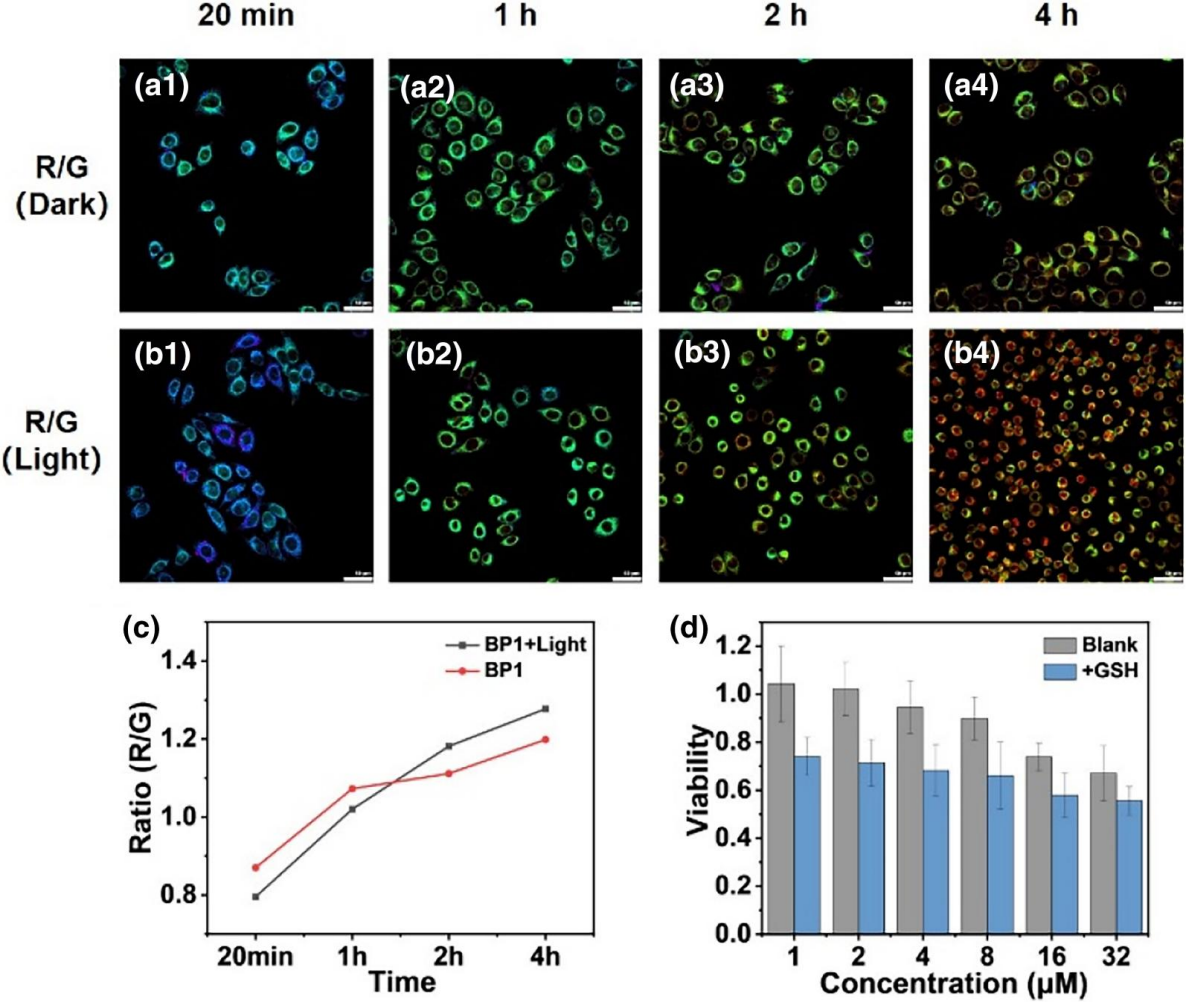
(a, b) Confocal ratio fluorescence image of HeLa cells incubated with **BP1 NPs** for different times; (a) Ratio of **BP1‐SG** channel to **BP1** channel under dark conditions; (b) Ratio of **BP1‐SG** channel to **BP1** channel under illumination; (c) Average fluorescence intensity ratio of the imaging results in (a, b); (d) The viability of Hela cell after treatment with various doses of **BP1**. (Gray column: blank group; Blue column: +GSH; White LED light, 40 mW/cm^2^) Ex: 487 nm, **BP1** channel, Em: 500–550 nm, **BP1‐SG** channel, Em: 570–620 nm. Scale bar: 50 μm.

Subsequently, to test whether the PDT process could aggravate the upregulation of GSH levels in cancer cells and promote the release of chemotherapeutic drugs, we compared the confocal images of HeLa cells incubated with **BP1 NPs** under light and dark conditions. As depicted in Figure 5a–c, as the incubation time was prolonged, the fluorescence intensity ratio between the red channel and the green channel (R/G) increased gradually under both light and dark conditions. From the confocal images, the color of the ratio image under illumination showed a more obvious variation than that without illumination. The R/G value increased from 0.79 to 1.28 under light conditions, while only increased from 0.87 to 1.19 under dark conditions. Similar experimental results were observed for **BP2 NPs** under identical conditions (Figure S12). The results indicated that the intracellular GSH concentration upon irradiation was higher than that in dark conditions, thus promoting the drug release process. We speculated that the light‐induced PDT process leads to the disruption of cellular homeostasis, thereby causing cellular oxidative stress. After PDT, cells improve the antioxidant capacity and alleviate the damage by upregulating a series of antioxidant substances such as GSH. Therefore, because of higher GSH concentrations, the reaction of **BP1 NPs** under light is faster than that in dark conditions, suggesting faster SN38 release.

To further demonstrate these results, we constructed two cell models with different GSH contents by co‐incubation with exogenous GSH for 1 h. CCK‐8 experiment was carried out to test the toxicity of **BP1 NPs** in the environment with different GSH levels. As shown in Figure 5d, cell viability decreased with the increase in **BP1** concentration in an evident dose‐dependent response. Importantly, incubating with the same concentration of **BP1 NPs**, cells with higher GSH levels had lower survival rates compared to untreated cells. In other words, high concentrations of GSH facilitate the substitution reaction, which can further release more SN38 and cause cell death. These results confirm that our design strategy not only provides a sequential photodynamic‐chemo combination theranostic system but also exhibits a photodynamic promoted chemotherapy process.

## 3 CONCLUSIONS

In conclusion, we have designed a sequential photodynamic‐chemo combination theranostic system with a tunable drug release rate. We have synthesized a series of BODIPY‐based prodrugs **BP1‐5**, in which the 3‐position of the BODIPY core is linked to a substituent with different electron‐withdrawing/donating abilities, and the 5‐position is linked to the chemotherapeutic drug SN38. The experimental results demonstrated that **BP** specifically responded to GSH and released SN38, and the reactivity of its nucleophilic substitution activity was regulated by adjusting the substituents, with an electron‐withdrawing group promoting the activity and an electron‐donating group reducing the activity. They are potentially more electron‐withdrawing/donating groups to be integrated to further regulate the drug release rate. The release process was also monitored in real time by fluorescence imaging. Moreover, **BP** molecules self‐assemble into nanoparticles, facilitating ROS generation for PDT. The PDT process also stimulated cells to passively upregulate GSH, further promoting the release of free SN38 for chemotherapy. The cytotoxicity experiments demonstrated that the effective killing of cancer cells was achieved by the combination of chemo‐photodynamic therapy. We hope this sequential photodynamic‐chemotherapy prodrug will provide a new platform and inspire more ideas for developing controllable drug delivery and theranostic systems.

## CONFLICT OF INTEREST STATEMENT

There are no conflicts to declare.

## ETHICS STATEMENT

No animal or human experiments were involved in this study.

## Supporting information

Supplementary Material

## Data Availability

The data that support the findings of this study are available in the supplementary material of this article.
