# Supplementary material for: Activatable theranostic prodrug scaffold with tunable drug release rate for sequential photodynamic and chemotherapy
Source: Smart Mol. 2024 Feb 20;2(1):e20230024. doi: 10.1002/smo.20230024 (PMC12118196; doi:10.1002/smo.20230024)
Supplement: Supplementary file 1 — Supplementary Material [file SMO2-2-e20230024-s001.docx]

Supporting Information

**Activable Theranostic Prodrug Scaffold with Tunable Drug Release Rate for Sequential Photodynamic and Chemotherapy**

Si-Yu Wang,^a^ Ying-Hao Pan,^a^ Yu-Chen Qu,^a^ Xiao-Xiao Chen,^a,b^ Na Shao,^a^ Li-Ya Niu ^a,^* and Qing-Zheng Yang ^a^

^a^Key Laboratory of Radiopharmaceuticals, College of Chemistry, Beijing Normal University, Beijing 100875, P. R. China.

^b^Department of Organic Chemistry, Universityof Geneva, 1211, Geneva, Switzerland

* E-mail: niuly@bnu.edu.cn

**Materials and instruments**

All of the reagents and solvents are from commercial suppliers and used without further purification. ^1^H and ^13^C NMR spectra were recorded with JEOL-400/600 spectrometers. High-resolution mass spectra were obtained on a Bruker Apex IV Fourier transform mass spectrometer. Fluorescence spectra were determined on a Hitachi F-4600 spectrophotometer, and absorption spectra were determined on a Hitachi UV-3900 spectrophotometer. Cell viability test was obtained on a Thermo Scientific Multiskan. Confocal fluorescence imaging was measured with Nikon A1R microscopy with a 60×oil-immersion objective lens. Emission was collected at 500-550 nm for the green channel and at 570-620 nm for the red channel (excited at 488 nm).

**Cell culture and fluorescence imaging**

HeLa, A549, HepG2 cells were cultured in culture media (DMEM/F12 supplemented with 10% FBS, 50 unit/mL penicillin, and 50 μg/mL of streptomycin) at 37 °C under a humidified atmosphere containing 5% CO_2_ for 24 h. The cells were seeded in a 6-well plate at a density of 104 cells per well in culture media. The cells were treated and incubated with 5 μM of **BP** molecules at 37 °C under 5 % CO_2_ for 15 min, then were washed three times with phosphate buffered saline (PBS). Confocal fluorescence imaging was performed with Nikon A1R microscopy. Green fluorescence was excited at 488 nm with a Si laser and emission was collected by a 500-550 nm band pass filter. Red fluorescence was excited at 488 nm and emission was collected by a 570-620 nm band pass filter.

**Cytotoxicity assay.**

The Cell Counting Kit-8 (CCK-8) cell proliferation assay was applied to investigate the cytotoxicity. Cells were seeded into a 96-well plate and incubated with 100 μL of Dulbecco’s modified Eagle medium (DMEM). After 24 h of cell attachment, the plates were washed with PBS, followed by the addition of increasing concentrations of probe 1 (0.5-16 μM) in DMEM. The cells were then incubated at 37℃ in an atmosphere of 5% CO_2_ and 95% air for 12 h, followed by standard CCK-8 assays (n = 6). Untreated assays (n = 6) were also conducted under the same conditions. The absorbance at 450 nm was measured by the Thermo Scientific Multiskan. Cell viability (%) = (Awith probe − Ablank/Acontrol − Ablank) × 100%.

**Synthesis**

Compound **A** and **B** were prepared according to the reported literature methods (See *Chem. Commun.*, **2019**, *55*, 13761-13764.).

Compound **A** (100 mg, 0.31 mmol) and 7-ethyl-10-hydroxycamptothecin (SN38, 149 mg, 0.38 mmol) were dissolved in 30 mL acetonitrile, followed by 3-4 drops of triethylamine, and the mixture was stirred at 50°C for 5 h. The mixed solution was washed with brine for 3 times and dried over Na_2_SO_4_. The crude residue was purified by column chromatography (SiO_2_, CH_2_Cl_2_/ethyl acetate = 1/2 as eluent) to give **BP1** (134 mg, 63%). ^1^H NMR (400 MHz, CDCl_3_): δ 8.29 (d, *J* = 9.2 Hz, 1H), 8.01 (d, *J* = 2.8 Hz, 1H), 7.82 (s, 1H), 7.76 (dd, *J* = 9.2, 2.5 Hz, 1H), 7.65 (s, 1H), 7.45 (d, *J* = 8.0 Hz, 2H), 7.31 (d, *J* = 7.8 Hz, 2H), 6.95 (d, *J* = 4.8 Hz, 1H), 6.82 (d, *J* = 4.1 Hz, 1H), 6.53 (m, 1H), 5.82 (d, *J* = 4.8 Hz, 1H), 5.75 (d, *J* = 16.5 Hz, 1H), 5.33 (d, *J* = 2.5 Hz, 1H), 5.28 (d, *J* = 6.1 Hz, 1H), 3.15 (q, *J* = 7.7 Hz, 2H), 2.46 (s, 3H), 1.89 (m, 2H), 1.46 – 1.34 (m, 3H), 1.04 (t, *J* = 7.3 Hz, 3H). ^13^C NMR (101 MHz, CDCl_3_): δ 173.94, 165.53, 157.71, 153.08, 152.41, 150.27, 147.65, 146.79, 145.54, 144.44, 140.97, 140.02, 134.16, 133.73, 133.18, 130.98, 130.71, 130.55, 130.01, 129.27, 128.92, 128.20, 127.88, 127.83, 124.05, 118.91, 116.96, 113.72, 105.28, 98.27, 72.86, 66.42, 49.48, 31.72, 30.65, 23.28, 21.51, 19.26, 14.15, 7.89. ESI-HRMS: [M+H]^+^ calculated 673.2434, found 673.2445.

Compound **B** (150 mg, 0.43 mmol) was dissolved in 30 mL anhydrous acetonitrile and sodium hydride (90 mg, 2.25 mmol) was added under agitation. 100 uL diethyl malonate (0.66 mmol) was slowly added to the mixture. After 2 h at rt, sodium hydride was quenched by slow drops of water. The reaction mixture was extracted with dichloromethane, dried over Na_2_SO_4_, and concentrated *in vacuo*. The crude residue was purified by column chromatography (SiO_2_, dichloromethane/petroleum ether = 2/1 as eluent) to give **2** (92 mg, 45%). ^1^H NMR (600 MHz, CDCl_3_): δ 7.96 (d, *J* = 8.8 Hz, 1H), 7.43 (d, *J* = 8.0 Hz, 1H), 7.34 (d, *J* = 8.1 Hz, 2H), 7.05 (d, *J* = 8.8 Hz, 2H), 6.96 (dd, *J* = 4.3, 1.1 Hz, 1H), 6.75 – 6.71 (m, 1H), 6.73 – 6.70 (m, 1H), 6.38 (d, *J* = 1.0 Hz, 1H),4.17 (m, 4H),3.34 (s, 1H), 2.44 (s, 3H), 1.27 (m, 6H).

Compound **2** (85 mg, 0.18 mmol) and 7-ethyl-10-hydroxy-camptothecin (SN38, 85 mg, 0.22 mmol) were dissolved in 30 mL acetonitrile with 3-4 drops of triethylamine and the mixture was stirred at 50°C for 5 h. The mixed solution was washed with brine for 3 times and dried over Na_2_SO_4_. The crude residue was purified by column chromatography (SiO_2_, dichloromethane/ethyl acetate = 1/1 as eluent) to give **BP2** (95 mg, 64%).^1^H NMR (600 MHz, CDCl_3_): δ 8.29 (d, *J* = 9.2 Hz, 1H), 7.97 (d, J = 2.5 Hz, 1H), 7.73 (d, *J* = 9.5 Hz, 1H), 7.66 (d, *J* = 2.0 Hz, 1H), 7.43 – 7.41 (m, 2H), 7.31 – 7.29 (m, 2H), 7.25 (s, 1H), 5.81 (dd, *J* = 4.7, 1.9 Hz, 1H), 5.73 (dd, *J* = 16.3, 1.9 Hz, 1H), 5.49 (d, *J* = 2.1 Hz, 1H), 5.33 – 5.26 (m, 2H), 3.15 (t, *J* = 8.0 Hz, 2H), 2.44 (s, 3H), 1.89 (m, 2H), 1.65 (s, 2H), 1.37 (td, *J* = 7.7, 1.9 Hz, 3H), 1.29 (m, 8H), 1.03 (td, *J* = 7.4, 1.9 Hz, 3H). ^13^C NMR (151 MHz, CDCl_3_): δ 173.95, 166.84, 165.37, 157.72, 153.08, 152.45, 150.28, 147.68, 146.80, 145.52, 144.17, 140.94, 133.81, 133.22, 130.53, 130.50, 129.25, 128.69, 127.88, 127.83, 123.97, 118.92, 118.06, 113.68, 105.29, 98.28, 62.28, 52.23, 49.47, 31.73, 29.38, 23.26, 21.49, 14.08, 7.87.ESI-HRMS: [M+H]^+^ calculated 831.3013, found 831.3000.

Compound **B** (800 mg, 2.29 mmol) was dissolved in 80 mL acetonitrile, followed by 3 mL ammonia and reaction at room temperature for 2 h. The mixed solution was washed with brine for 3 times and dried over Na_2_SO_4_. The crude residue was purified by column chromatography over silica (dichloromethane/petroleum ether = 5/1 as eluent) to give **3-1** (285 mg, 38%). ^1^H NMR (600 MHz, CDCl_3_): δ 7.96 (d, *J* = 8.8 Hz, 1H), 7.45 (d, J = 8.0 Hz, 1H), 7.32 (d, *J* = 8.1 Hz, 2H), 7.04 (d, *J* = 8.8 Hz, 2H), 6.96 (dd, *J* = 4.3, 1.1 Hz, 1H), 6.76 – 6.73 (m, 1H), 6.74 – 6.71 (m, 1H), 6.36 (d, *J* = 1.0 Hz, 1H), 5.33 (s, 2H), 2.45 (s, 3H).

Compound **3-1** (200 mg, 0.60 mmol) was dissolved in 50 mL acetonitrile, with 0.5 mL drops of triethylamine, and placed in an ice water bath. Stir and slowly drop 100 μL acetyl bromide and continue to react at room temperature for 3 h. The mixed solution was washed with brine for 3 times and dried over Na_2_SO_4_. The crude residue was purified by column chromatography over silica (dichloromethane/petroleum ether = 3/1 as eluent) to give **3-2** (119 mg, 53%). ^1^H NMR (600 MHz, CDCl_3_): δ 7.95 (d, *J* = 8.8 Hz, 1H), 7.47 (d, *J* = 8.0 Hz, 1H), 7.32 (d, *J* = 8.1 Hz, 2H), 7.05 (d, *J* = 8.8 Hz, 2H), 6.98 (dd, *J* = 4.3, 1.1 Hz, 1H), 6.79 – 6.77 (m, 1H), 6.76 – 6.74 (m, 1H), 6.35 (d, *J* = 1.0 Hz, 1H), 6.71 (s, 1H), 2.46 (s, 3H), 2.25 (s, 3H).

Compound **3-2** (110 mg, 0.29 mmol) and 7-ethyl-10-hydroxycamptothecin (SN38, 170 mg, 0.43 mmol) were dissolved in 30 mL acetonitrile with 3-4 drops of triethylamine and reacted at 50°C for 5 h. The mixed solution was washed with brine for 3 times and dried over Na_2_SO_4_. The crude residue was purified by column chromatography over silica (dichloromethane/ethyl acetate = 1/1 as eluent) to give **BP3** (88 mg, 37%). ^1^H NMR (600 MHz, CDCl_3_): δ 8.70 (s, 1H), 8.27 (d, J = 9.1 Hz, 1H), 7.93 (s, 1H), 7.76 – 7.68 (m, 1H), 7.65 (s, 1H), 7.51 (d, *J* = 5.5 Hz, 1H), 7.41 (d, *J* = 7.6 Hz, 2H), 7.30 (d, *J* = 7.8 Hz, 2H), 7.25 (s, 1H), 7.15 (d, *J* = 4.6 Hz, 1H), 6.90 (s, 2H), 6.74 (s, 1H), 5.73 – 5.69 (m, 1H), 5.31 (s, 1H), 5.27 (d, *J* = 11.2 Hz, 2H), 3.13 (d, *J* = 8.0 Hz, 2H), 2.44 (s, 3H), 2.27 (s, 3H), 1.89 (dd, *J* = 18.0, 8.0 Hz, 3H), 1.37 (t, *J* = 7.7 Hz, 3H), 1.02 (t, *J* = 7.3 Hz, 3H). ^13^C NMR (151 MHz, CDCl_3_): δ 173.95, 167.77, 161.42, 157.76, 153.86, 152.10, 150.26, 150.12, 147.42, 146.87, 145.25, 141.36, 140.65, 133.05, 132.58, 130.97, 130.94, 130.48, 129.34, 129.25, 128.92, 128.88, 127.89, 127.72, 123.85, 118.82, 112.75, 111.75, 101.84, 98.17, 72.86, 49.48, 31.75, 30.66, 24.61, 23.24, 21.47, 19.25, 14.16, 14.09, 13.78, 7.88.ESI-HRMS: [M+H]^+^ calculated 730.2648, found 730.2640.

Compound **B** (120 mg, 0.34 mmol), Na_2_CO_3_ (180 mg, 1.70 mmol) and tetra-(triphenylphosphine) palladium (20mg, 17.3 μmol) were dissolved in 30 mL toluene. Phenylboric acid (42 mg, 0.34 mmol) dissolved in 10 mL toluene solution was added using a syringe under nitrogen protection and reacted at 80°C for 3 h. 150 mL brine was added into the mixed solution and extracted with dichloromethane. The organic phase was dried with Na_2_SO_4_ and filtered and concentrated *in vacuo* to get crude products. The crude residue was purified by column chromatography over silica (petroleum ether/ethyl acetate = 20/1 as eluent) to give **4-1** (62 mg, 46%). ^1^H NMR (600 MHz, CDCl_3_): δ 7.97 (d, *J* = 8.8 Hz, 1H), 7.45 (d, *J* = 8.0 Hz, 1H), 7.41 (t, *J* = 7.3 Hz, 1H),7.33 (d, *J* = 8.1 Hz, 2H), 7.01 (d, *J* = 8.8 Hz, 2H), 6.91 (dd, *J* = 4.3, 1.1 Hz, 1H), 6.74 – 6.76 (m, 1H), 6.70 (m, 1H), 6.33 (d, *J* = 1.0 Hz, 1H), 2.45 (s, 3H).

Compound **4-1** (50 mg, 0.13 mmol) and 7-ethyl-10-hydroxycamptothecin (SN38, 70 mg, 0.18 mmol) were dissolved in 20 mL acetonitrile with 3-4 drops of triethylamine and reacted at 50°C for 5 h. The mixed solution was washed with brine for 3 times and dried over Na_2_SO_4_. The crude residue was purified by column chromatography over silica (dichloromethane/ethyl acetate = 4/1 as eluent) to give **BP4** (53 mg, 56%). ^1^H NMR (600 MHz, CDCl_3_): δ 8.27 (d, *J* = 9.2 Hz, 1H), 7.93 (d, *J* = 2.8 Hz, 1H), 7.93 – 7.90 (m, 2H), 7.71 (d, *J* = 2.6 Hz, 1H), 7.67 (d, *J* = 1.0 Hz, 1H), 7.47 (dd, *J* = 8.2, 1.9 Hz, 2H), 7.45 – 7.43 (m, 2H), 7.40 (t, *J* = 7.3 Hz, 1H), 7.33 (d, *J* = 7.8 Hz, 2H), 6.88 (d, *J* = 4.6 Hz, 1H), 6.87 (d, *J* = 4.3 Hz, 1H), 6.61 (d, *J* = 4.3 Hz, 1H), 5.81 (d, *J* = 4.6 Hz, 1H), 5.72 (d, *J* = 16.3 Hz, 1H), 5.29 (d, *J* = 16.3 Hz, 1H), 5.25 (s, 2H), 3.11 (q, *J* = 7.7 Hz, 2H), 2.47 (s, 3H), 1.94 – 1.84 (m, 3H), 1.35 (t, *J* = 7.7 Hz, 3H), 1.02 (t, *J* = 7.4 Hz, 3H). ^13^C NMR (151 MHz, CDCl_3_): δ 173.93, 164.44, 157.74, 156.80, 153.39, 152.23, 150.36, 147.51, 146.81, 145.52, 143.40, 140.72, 135.88, 133.05, 132.26, 130.94, 130.64, 129.72, 129.41, 129.27, 129.23, 128.33, 127.89, 127.78, 124.03, 119.52, 118.88, 113.47, 104.87, 98.34, 72.92, 66.40, 49.51, 31.75, 29.79, 29.42, 23.26, 21.54, 14.12, 7.91. ESI-HRMS: [M+H]^+^ calculated 749.2741, found 749.2741.


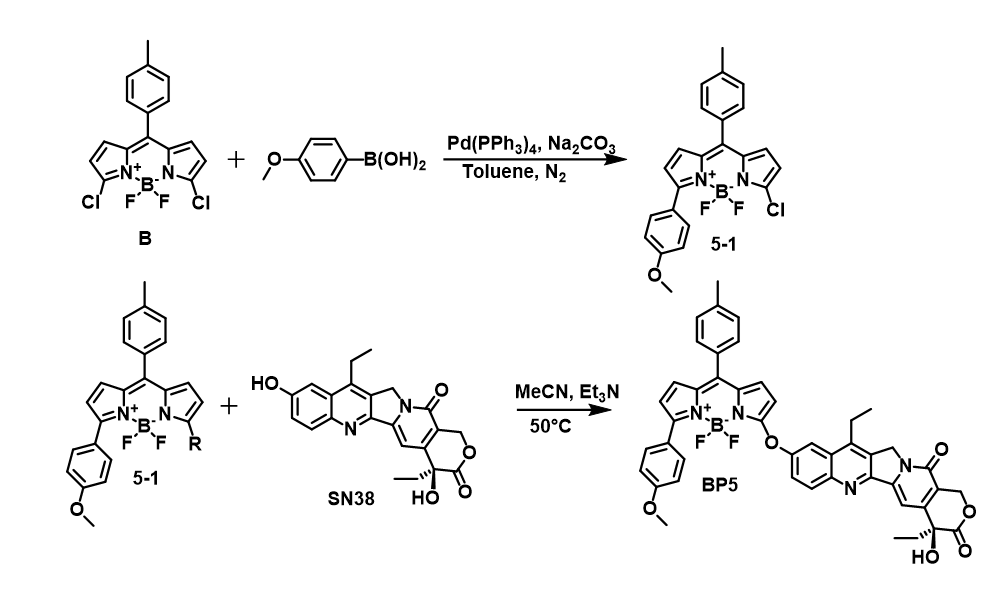


Compound **B** (120 mg, 0.34 mmol), Na_2_CO_3_ (180 mg, 1.70 mmol) and tetra-(triphenylphosphine) palladium (20 mg, 17.3 μmol) were dissolved in 30 mL toluene. P-methoxyphenylboric acid (52 mg, 0.34 mmol) dissolved in 10 mL toluene was added using a syringe under nitrogen protection and reacted at 80°C for 3 h. 150 mL brine was added to the mixed solution and extracted with dichloromethane. The organic phase was dried with Na_2_SO_4_ and filtered and concentrated *in vacuo* to get crude products. The crude residue was purified by column chromatography over silica (petroleum ether/ethyl acetate = 20/1 as eluent) to give **5-1** (55 mg, 38%). ^1^H NMR (600 MHz, CDCl_3_): δ 7.98 (d, *J* = 8.8 Hz, 1H), 7.43 (d, *J* = 8.0 Hz, 1H), 7.32 (d, *J* = 8.1 Hz, 2H), 7.02 (d, *J* = 8.8 Hz, 2H), 6.94 (dd, *J* = 4.3, 1.1 Hz, 1H), 6.76 – 6.75 (m, 1H), 6.71 – 6.70 (m, 1H), 6.36 (d, *J* = 1.0 Hz, 1H), 3.88 (s, 3H), 2.47 (s, 3H).

Compound **5-1** (45 mg, 0.11 mmol) and 7-ethyl-10-hydroxycamptothecin (SN38, 80 mg, 0.20 mmol) were dissolved in 40 mL acetonitrile with 3-4 drops of triethylamine and reacted at 50°C for 5 h. The mixed solution was washed with brine for 3 times and dried over Na_2_SO_4_. The crude residue was purified by column chromatography over silica (dichloromethane/ethyl acetate = 4/1 as eluent) to give **BP5** (51 mg, 61%). ^1^H NMR (400 MHz, CDCl_3_): δ 8.26 (d, *J* = 9.1 Hz, 1H), 7.92 (d, *J* = 2.4 Hz, 1H), 7.90 (s, 1H), 7.70 (dd, *J* = 9.2, 2.6 Hz, 1H), 7.67 (d, *J* = 1.0 Hz, 1H), 7.45 (d, *J* = 8.1 Hz, 2H), 7.31 (d, *J* = 7.9 Hz, 2H), 6.96 (d, *J* = 9.0 Hz, 2H), 6.84 (dd, *J* = 8.1, 4.4 Hz, 2H), 6.60 (d, *J* = 4.3 Hz, 1H), 5.79 (dd, *J* = 4.5, 0.7 Hz, 1H), 5.70 (d, *J* = 16.4 Hz, 1H), 5.29 (d, *J* = 6.7 Hz, 1H), 5.25 (d, *J* = 7.4 Hz, 2H), 3.82 (s, 3H), 3.11 (q, *J* = 7.6 Hz, 2H), 2.46 (s, 3H), 1.95 – 1.80 (m, *J* = 7.2 Hz, 2H), 1.35 (t, *J* = 7.6 Hz, 3H), 1.01 (t, *J* = 7.4 Hz, 3H). ^13^C NMR (101 MHz, CDCl_3_): δ 173.88, 163.65, 160.72, 157.71, 157.21, 153.57, 152.07, 150.35, 147.38, 146.77, 145.47, 142.65, 140.56, 136.02, 132.95, 131.27, 131.02, 130.99, 130.62, 130.17, 129.17, 128.90, 127.88, 127.74, 125.31, 124.01, 119.55, 118.82, 113.91, 113.24, 104.23, 98.35, 72.93, 66.35, 55.38, 49.50, 31.71, 23.24, 21.52, 14.10, 7.91. [M+H]^+^ calculated 779.2847, found 779.2845.


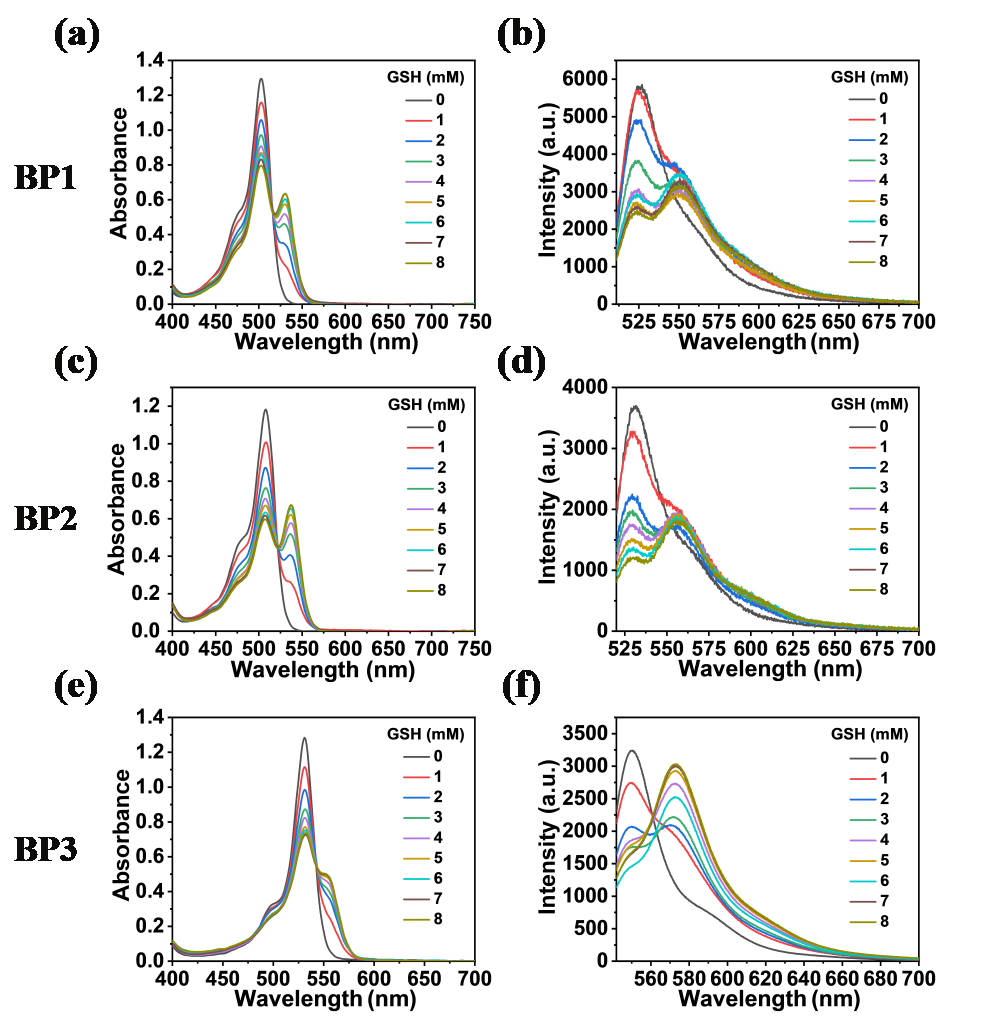


**Figure S1**. (a, c, e) Absorption spectra of 10 μM **BP1-3** upon the addition of 0 - 8 mM GSH. (b, h, f) Fluorescence spectra of 10 μM **BP1-3** upon the addition of 0 - 8 mM GSH.


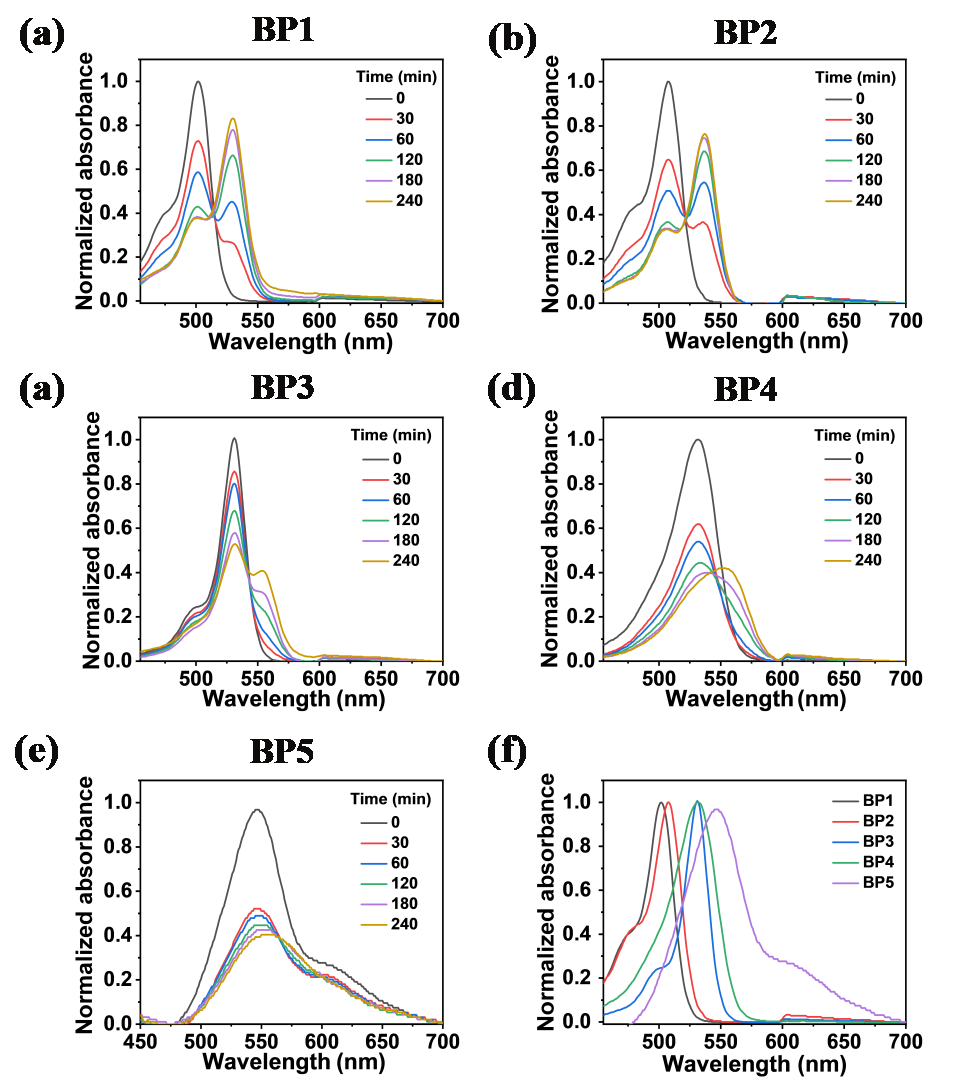


**Figure S2**. (a-e) Time-dependent absorption spectra of 10 μM **BP1-5** treated with 1 mM GSH. (f) Normalized absorption spectra of **BP** molecules.


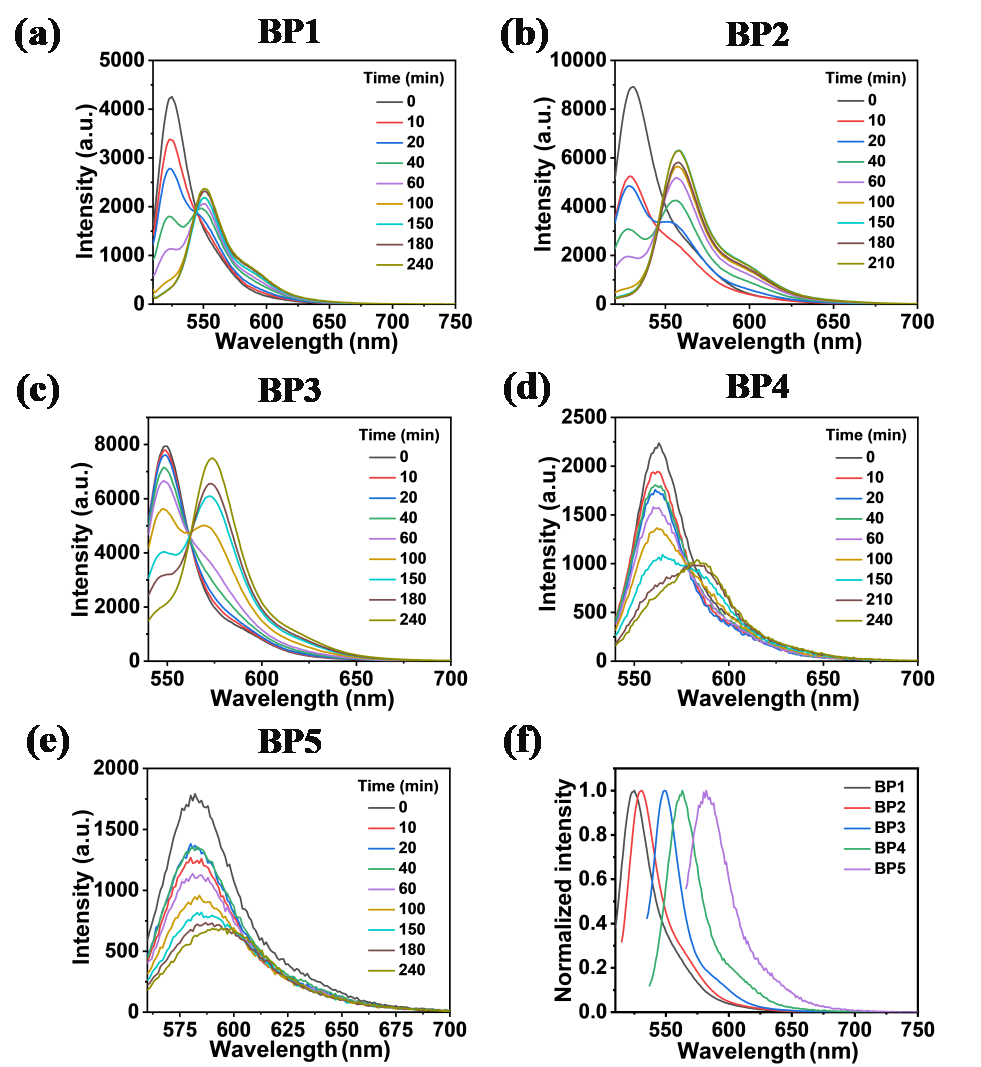


**Figure S3**. (a-e) Time-dependent fluorescence spectra of 10 μM **BP1-5** treated with 1 mM GSH. (f) Normalized fluorescence spectra of **BP** molecules.


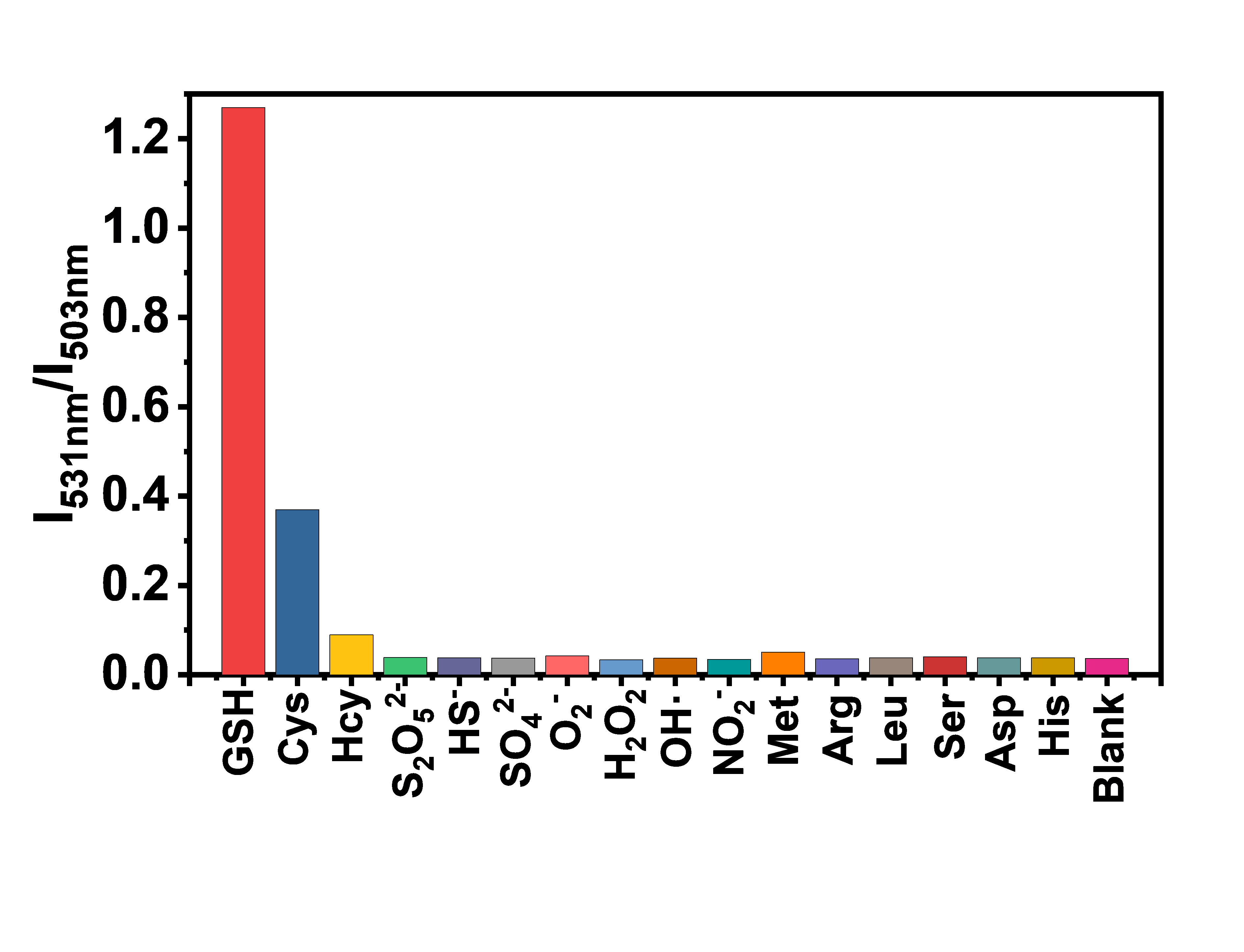


**Figure S4**. Absorption ratio of probe **BP1** in the presence of other biothiols and reagents.


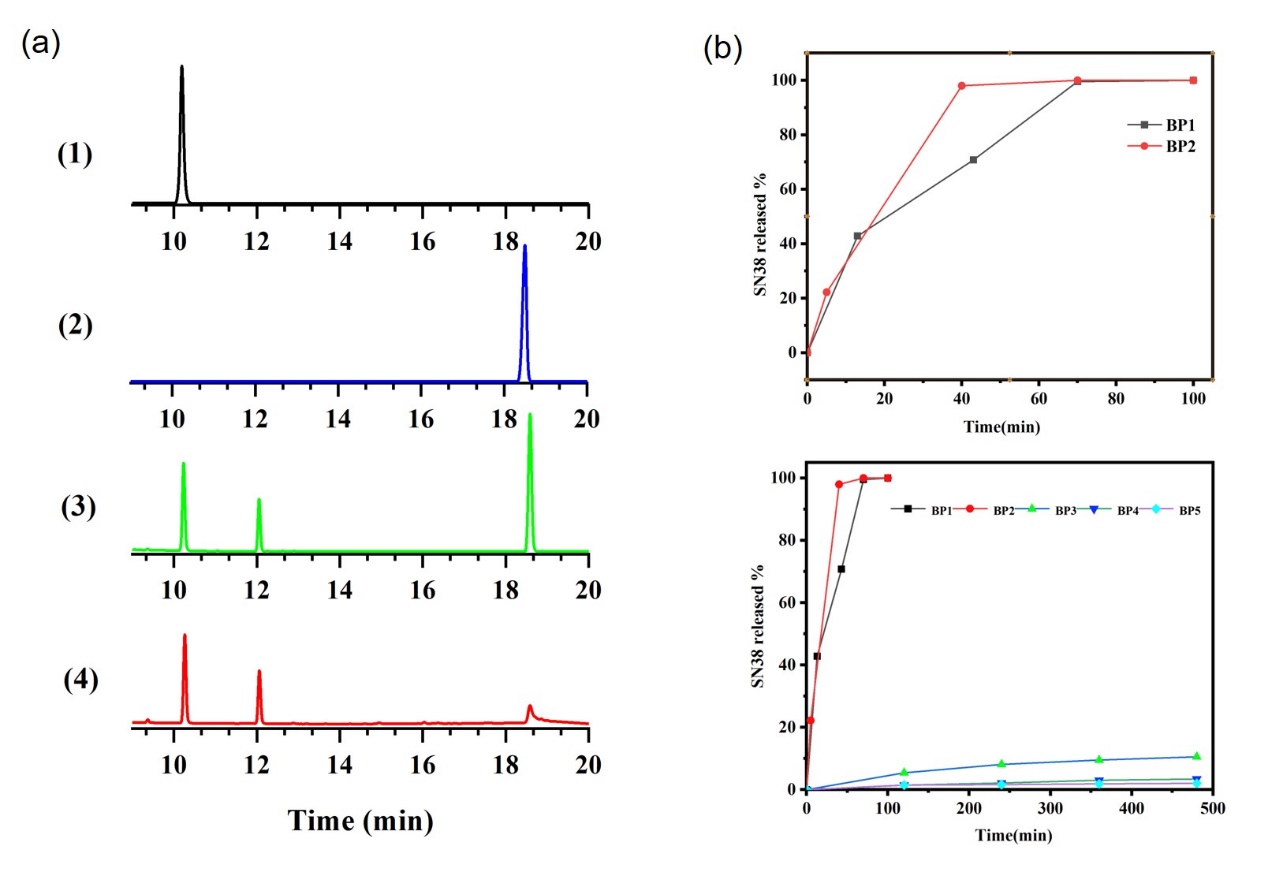


**Figure S5**. (a) HPLC analysis of the solution of (1) SN-38, (2) **BP1**, (3) **BP1** + GSH at 10 min; (4) **BP1** + GSH at 100 min. (b) The SN-38 release kinetics of **BP1-5.**

**Figure S6**. (a-e) Ratio of the maximum absorption intensity of **BP** molecules before and after reaction with different concentrations of GSH.


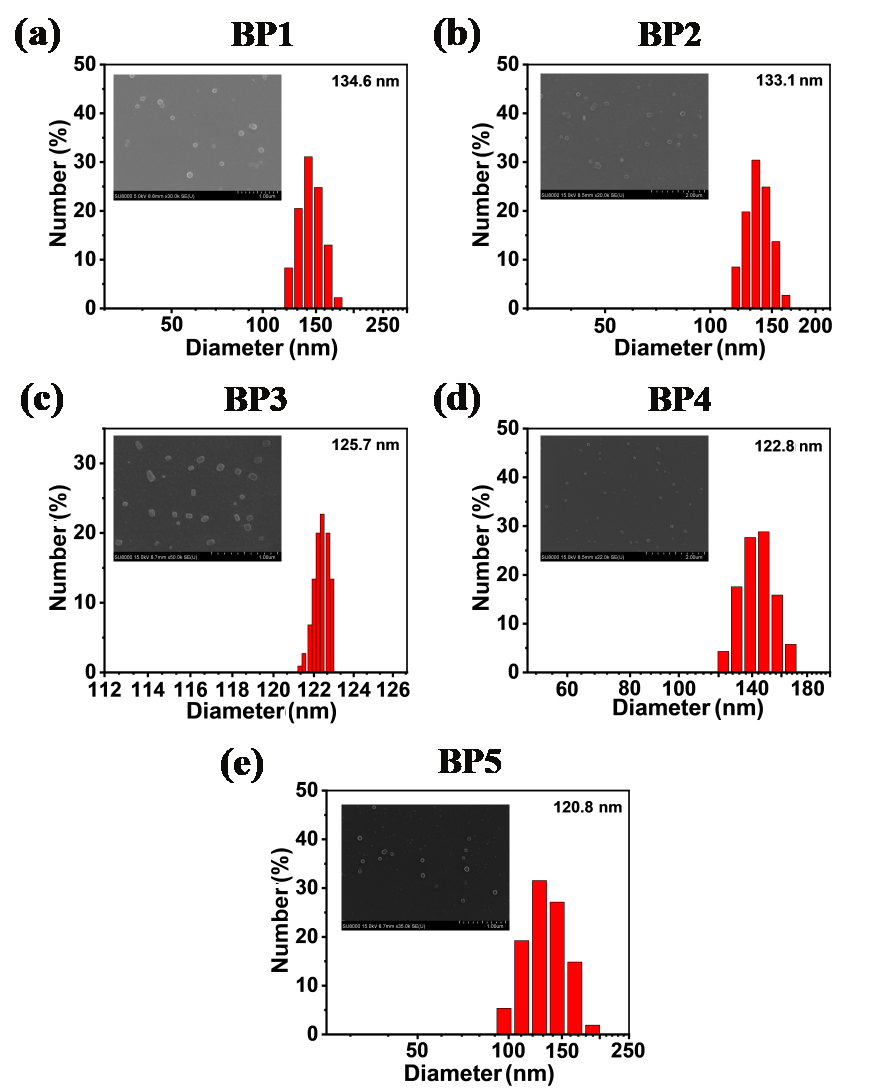


**Figure S7**. (a-e) Size distribution of **BP1-5 NPs** using DLS (inset: SEM image of **BP1-5 NPs**).


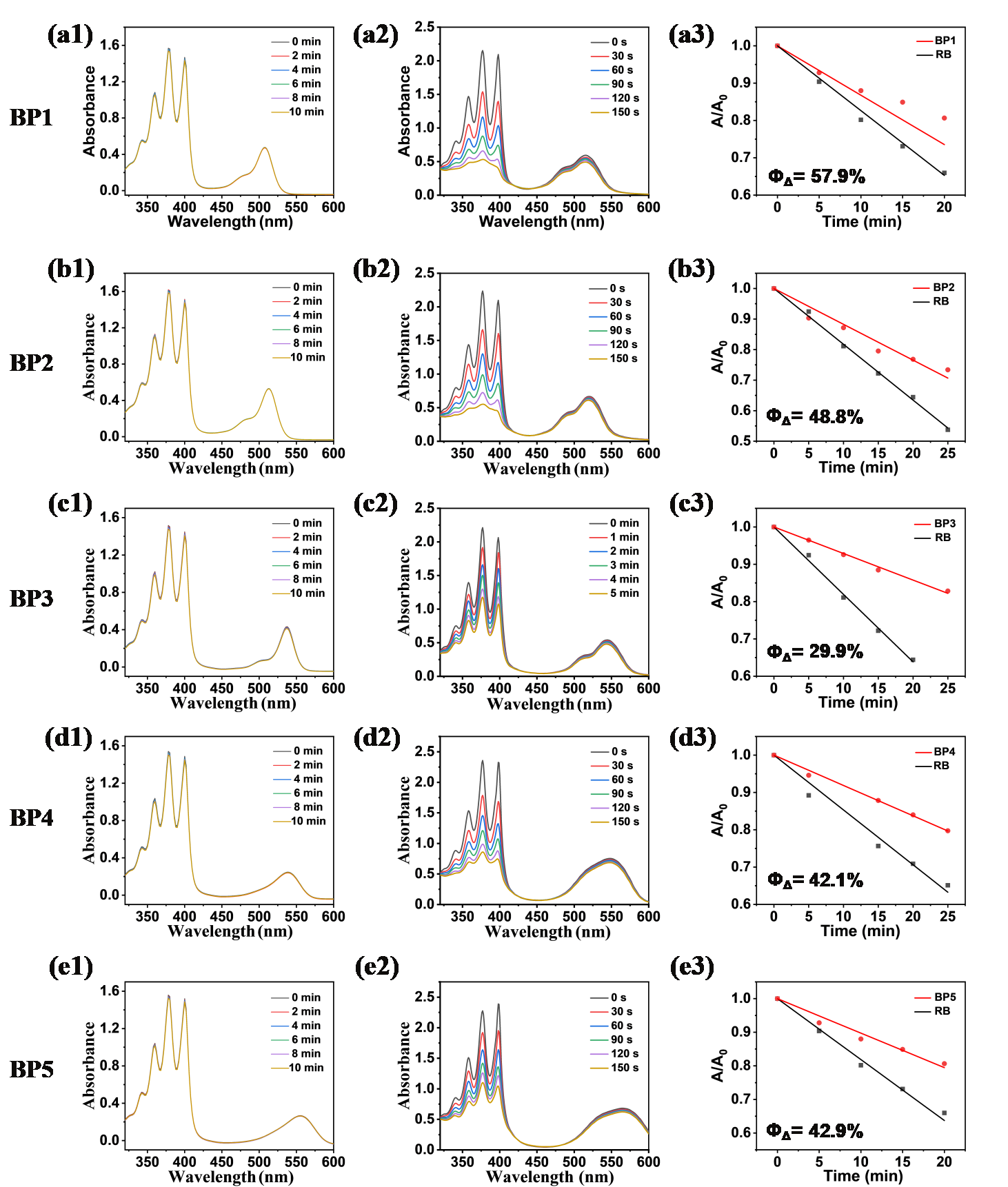


**Figure S8**. (a1-e1) Absorption spectra of ABDA in the presence of **BP1-5** in MeCN upon light irradiation for different time. (a2-e2) Absorption spectra of ABDA and **BP1-5 NPs** (in aqueous dispersion solution) upon light irradiation for different time. (a3-e3) The absorbance changes of ABDA at 378 nm in the presence of **BP 1-5 NPs** and RB.


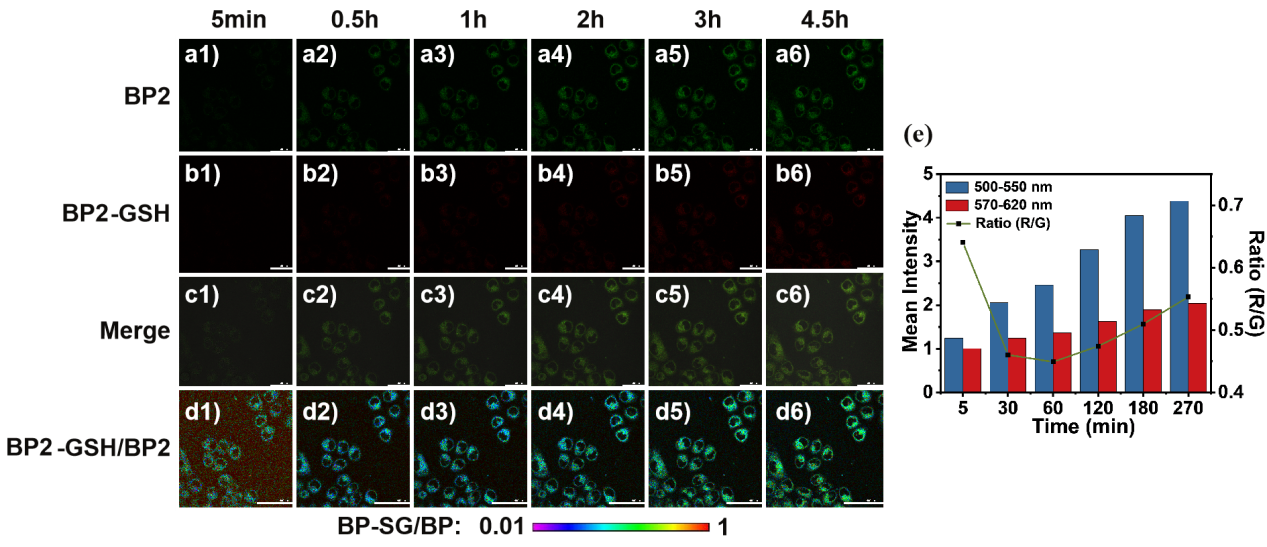


**Figure S9.** (a-d) Confocal fluorescence image of HeLa cell incubated with **BP2** for different time; (a) **BP2** channel; (b) **BP2-SG** channel; (c) Merge; (d) Ratio of **BP2-SG** channel to **BP2** channel. Scale bar: 50 μm or 100 μm.


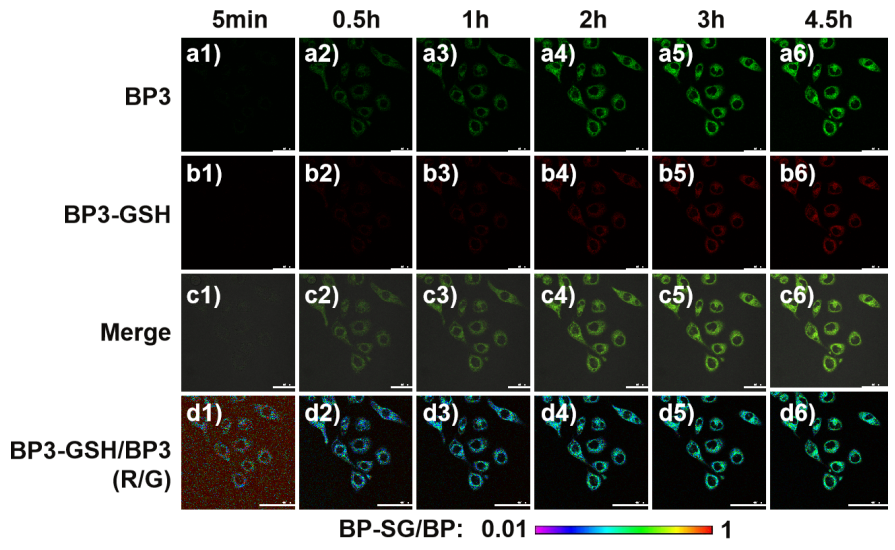


**Figure S10.** (a-d) Confocal fluorescence image of HeLa cell incubated with **BP3** for different time; (a) **BP3** channel; (b) **BP3-SG** channel; (c) Merge; (d) Ratio of **BP3-SG** channel to **BP3** channel. Scale bar: 50 μm or 100 μm.


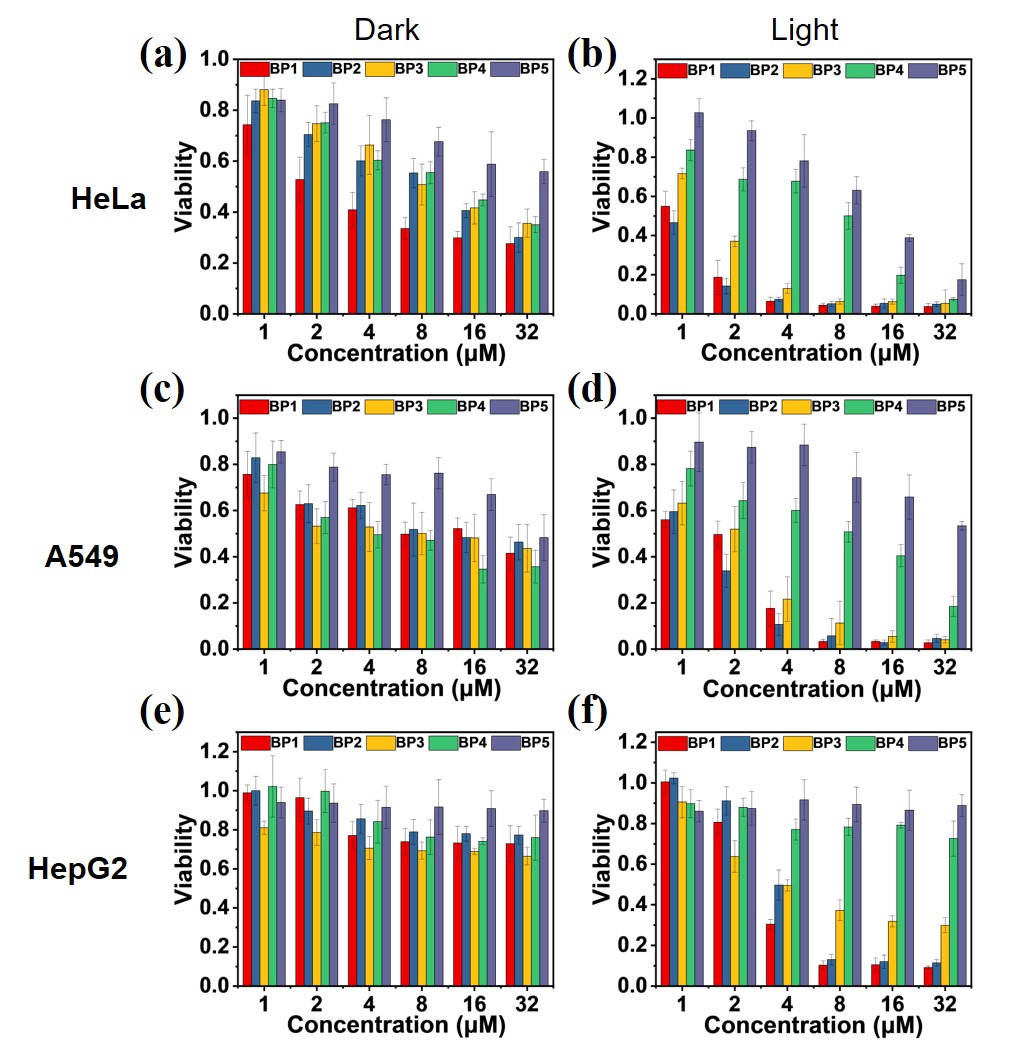


**Figure S11.** Cell viability of HeLa cells subjected to a range of **BP1-5** (a) in the dark and (b) upon light irradiation (White LED light, 40 mW/cm^2^)


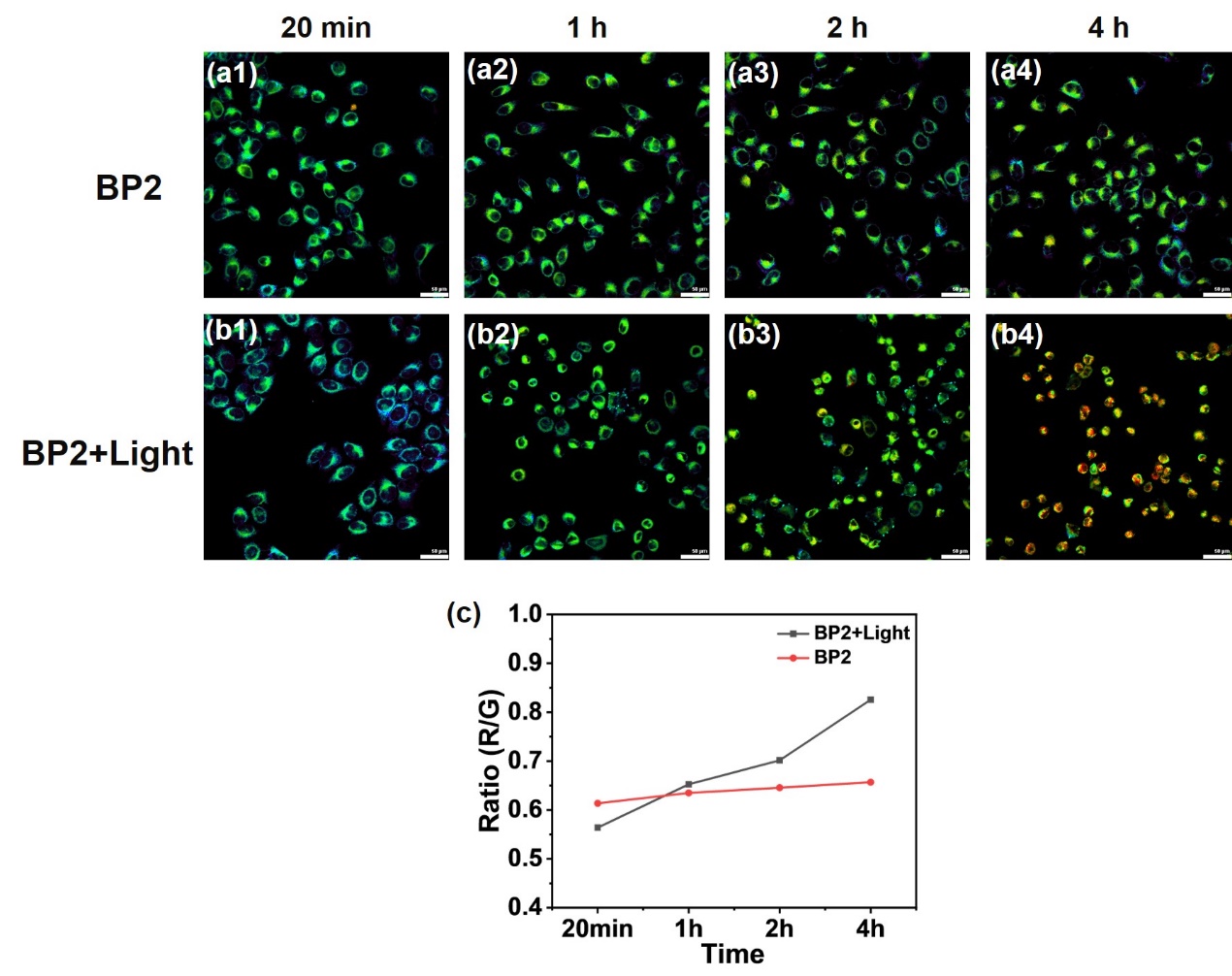


**Figure S12.** (a-b) Confocal fluorescence ratio image of HeLa cell incubated with **BP2** for different time; (a) Ratio of **BP2-SG** channel to **BP2** channel without illumination; (b) Ratio of **BP2-SG** channel to **BP2** channel after illumination; (c) Average fluorescence intensity of the imaging results in (a-b). Ex: 487 nm, **BP2** channel Em: 500-550 nm, **BP2-SG** channel Em: 570-620 nm. Scale bar: 50 μm.

**^1^H NMR, ^13^C NMR and HRMS spectra of BP 1-5**


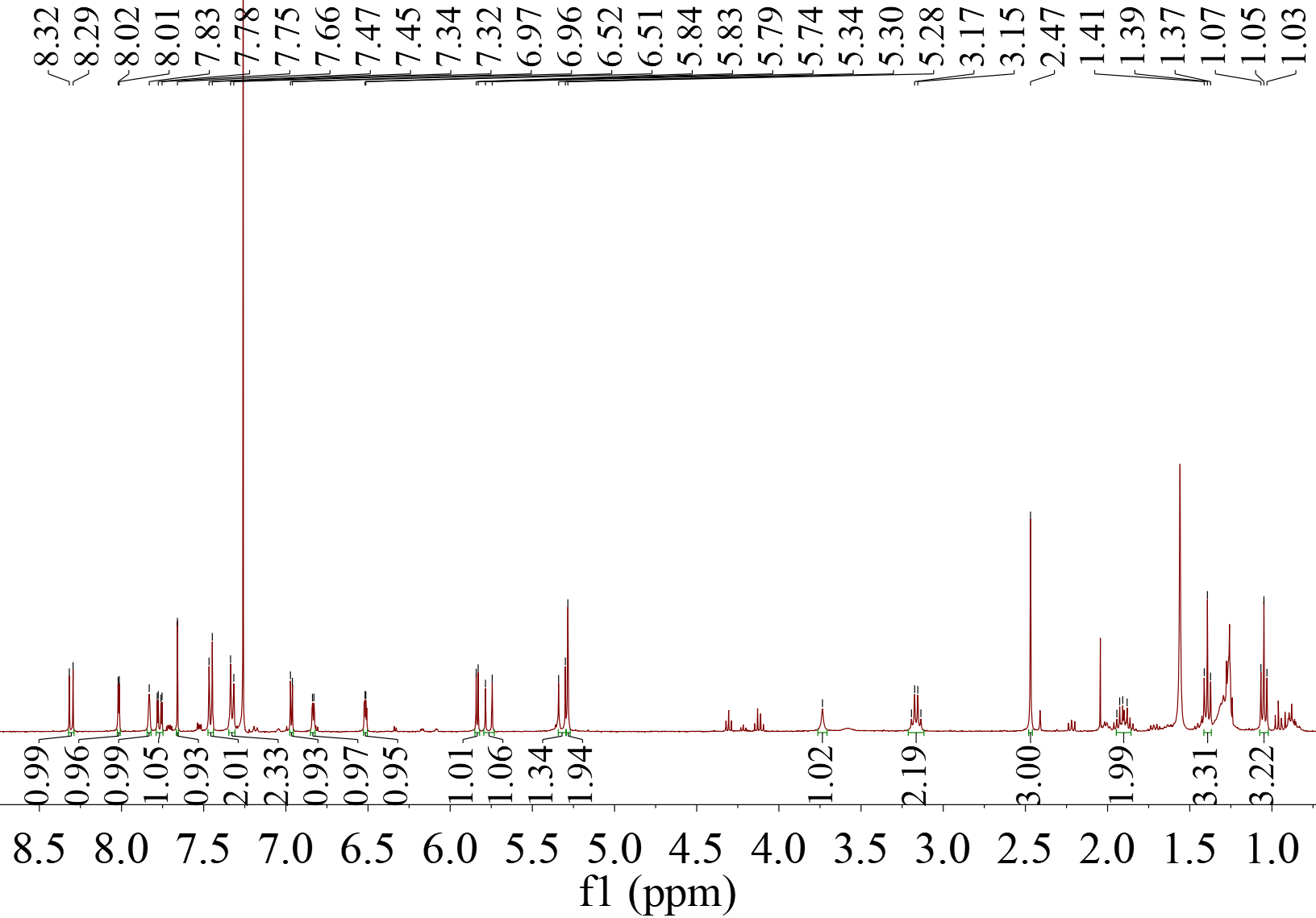


^1^H NMR spectra of **BP1**


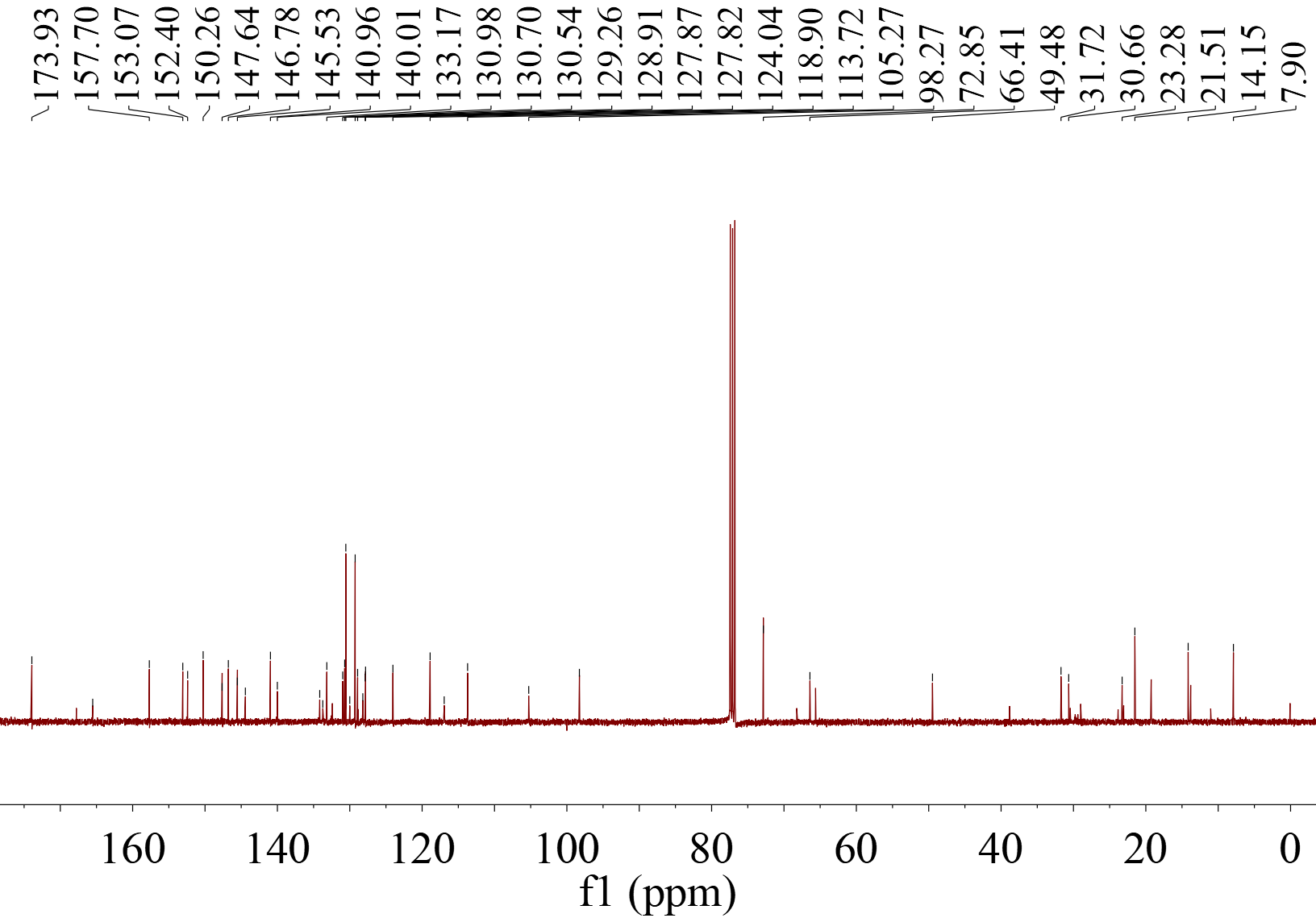


^13^C NMR spectra of **BP1**


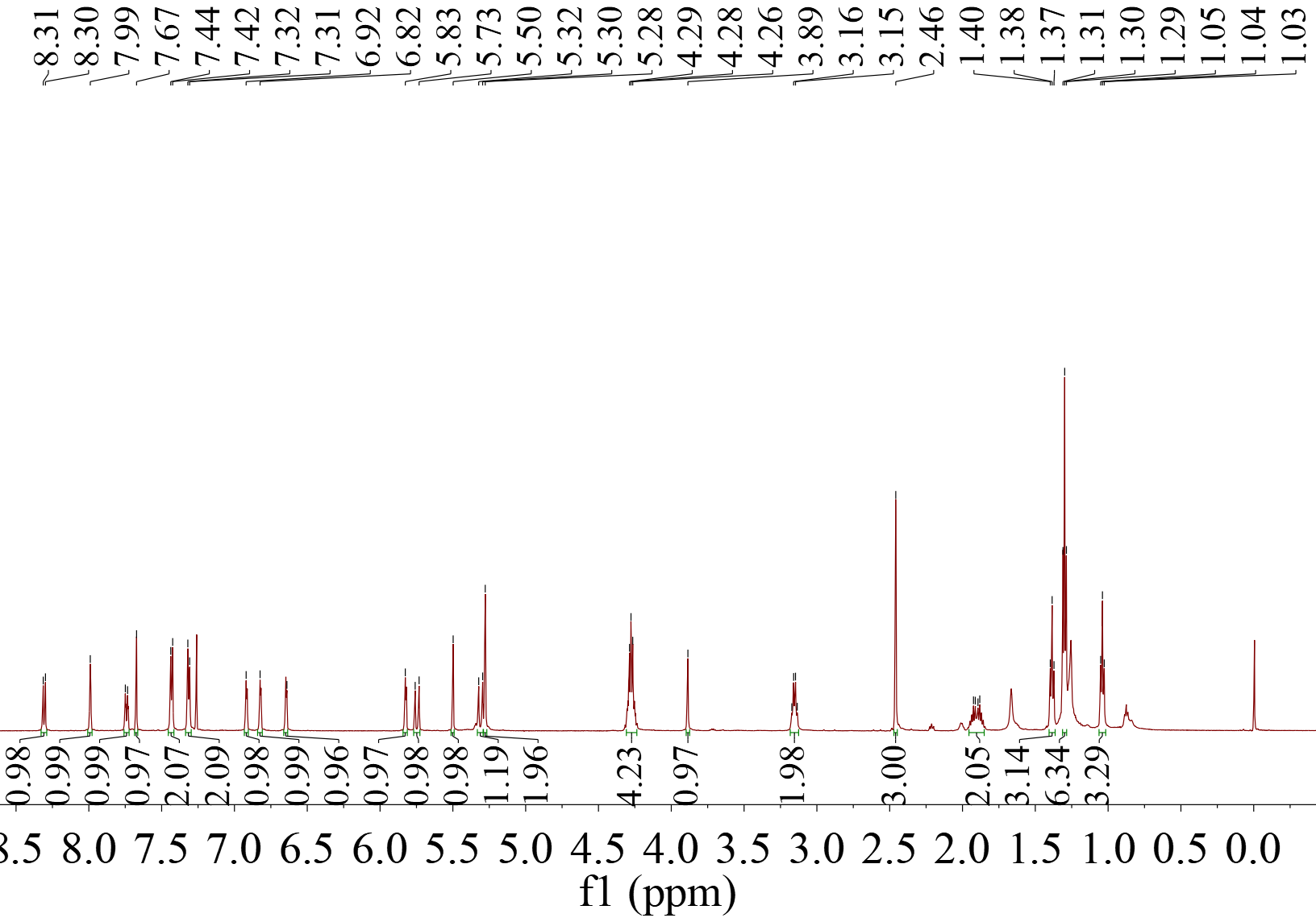


^1^H NMR spectra of **BP2**


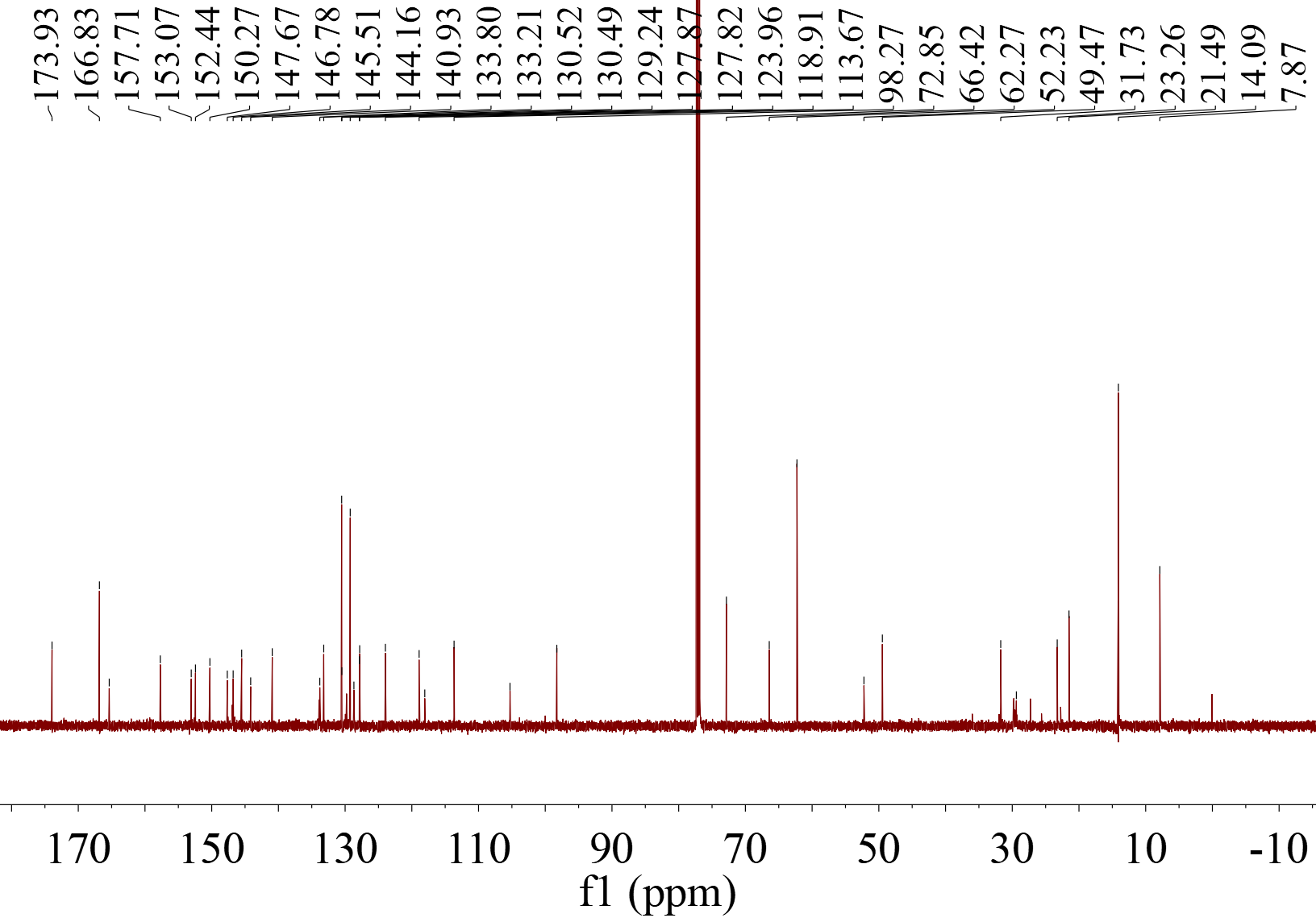


^13^C NMR spectra of **BP2**


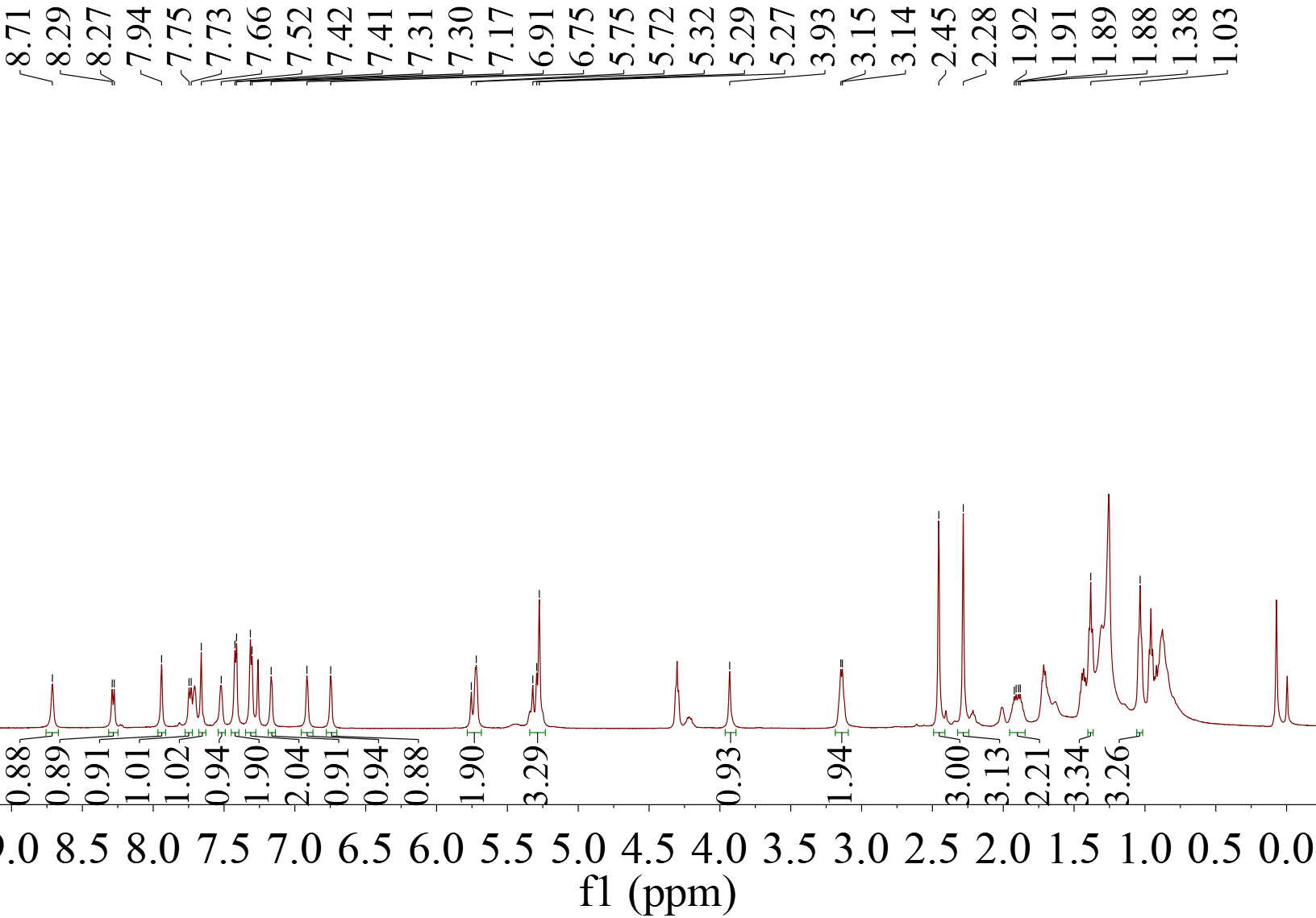


^1^H NMR spectra of **BP3**


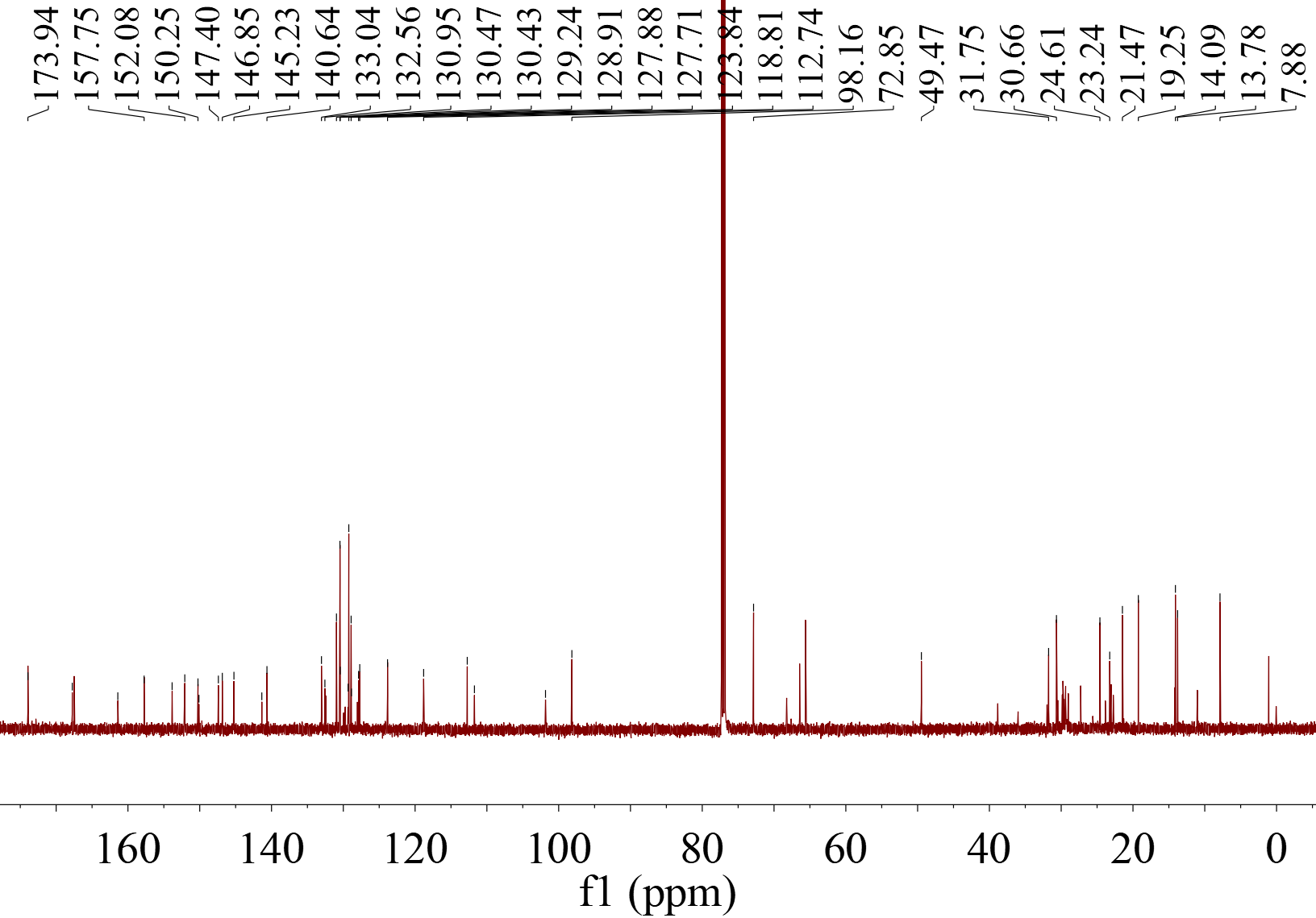


^13^C NMR spectra of **BP3**


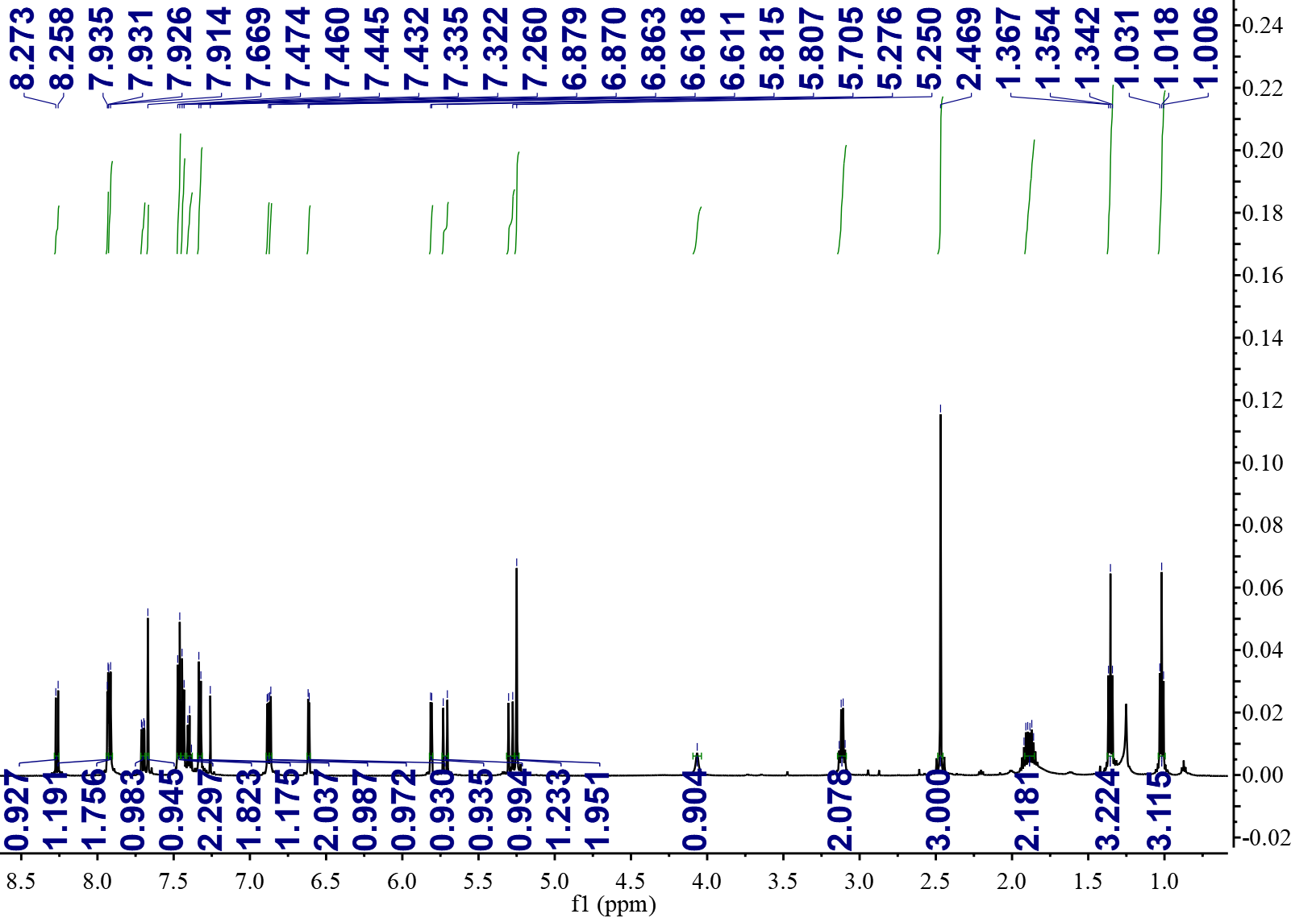


^1^H NMR spectra of **BP4**


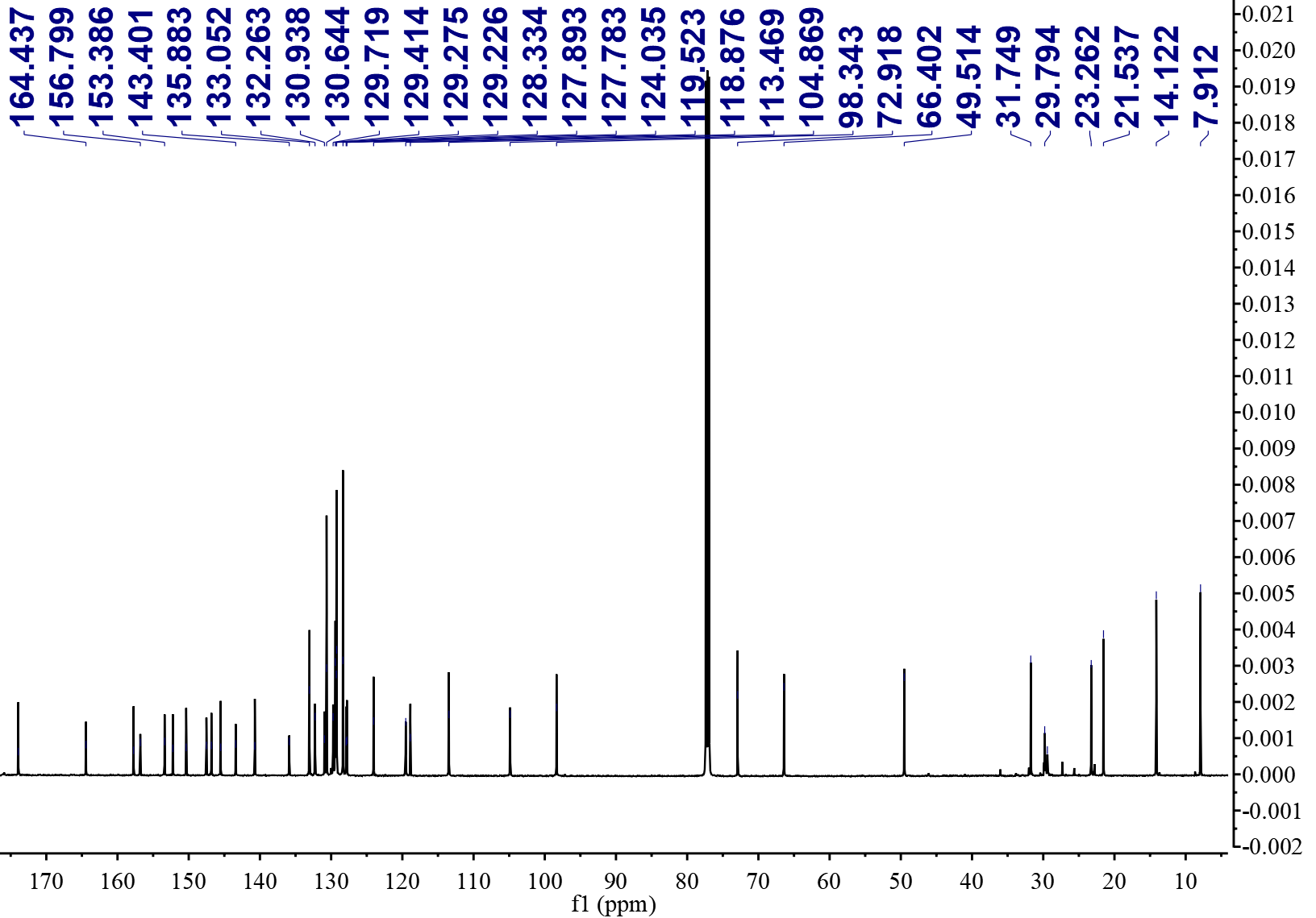


^13^C NMR spectra of **BP4**


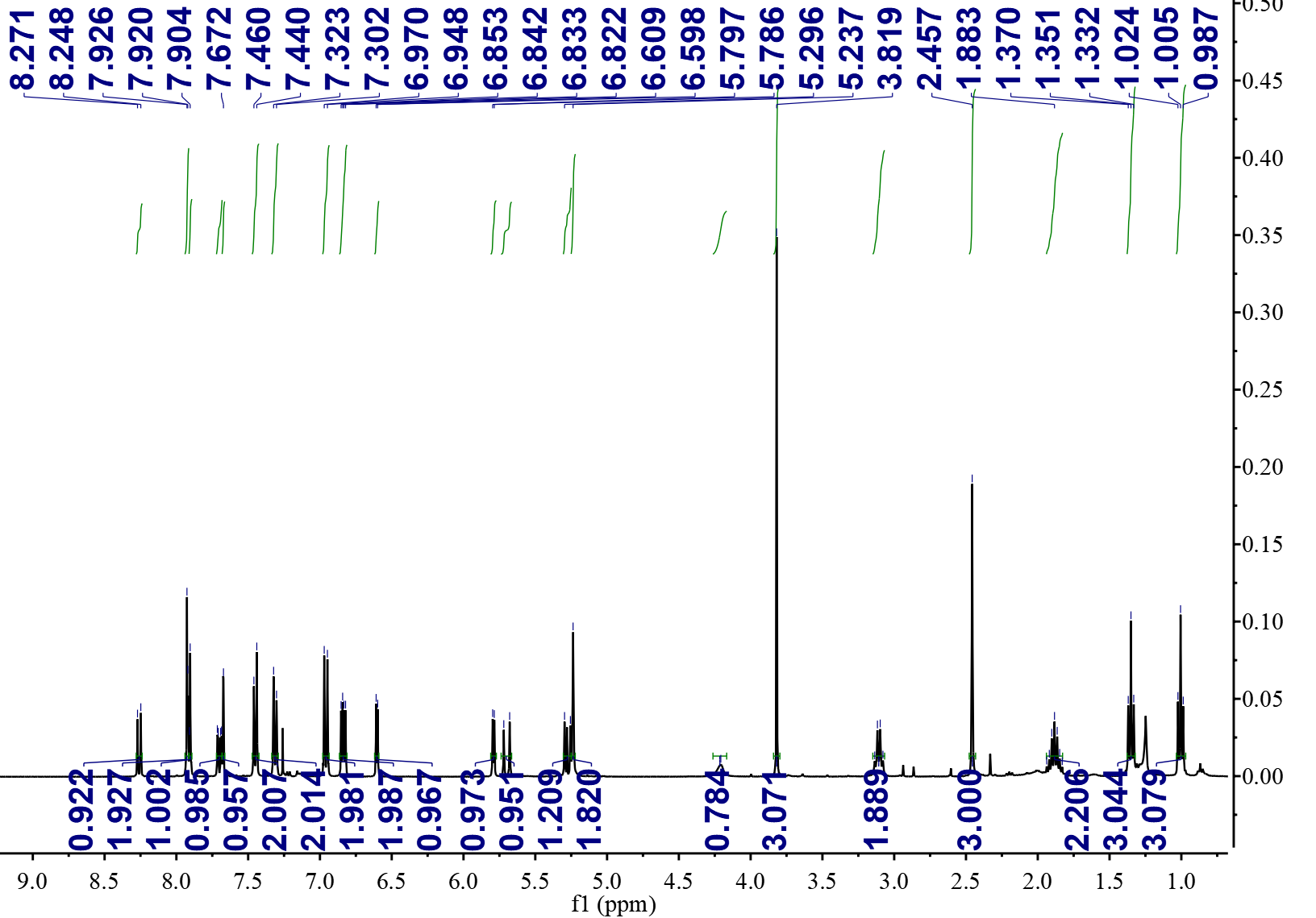


^1^H NMR spectra of **BP5**


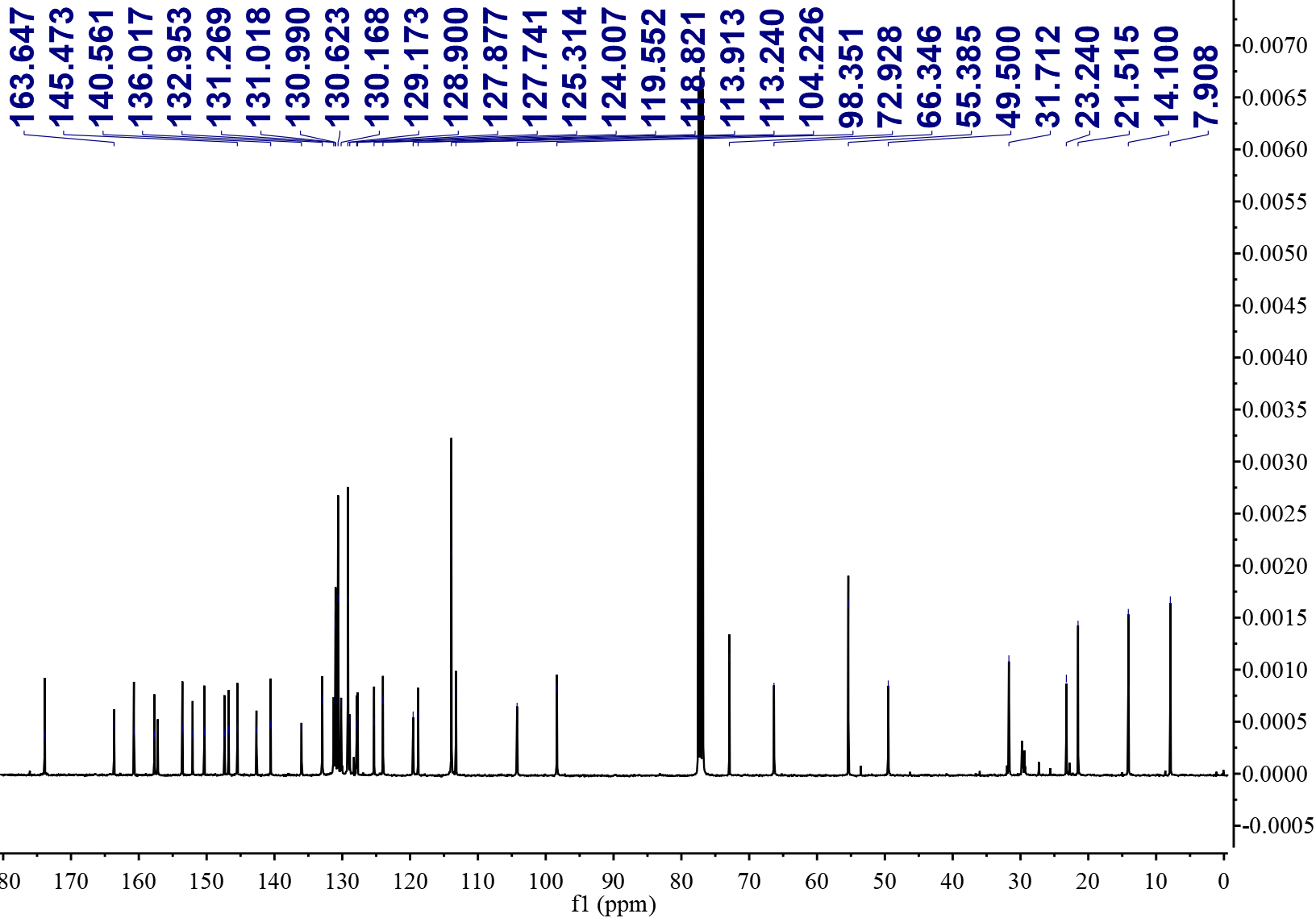


^13^C NMR spectra of **BP5**
